# BAP1 induces cell death via interaction with 14-3-3 in neuroblastoma

**DOI:** 10.1038/s41419-018-0500-6

**Published:** 2018-04-24

**Authors:** Wondossen Sime, Qiankun Niu, Yasmin Abassi, Katarzyna Chmielarska Masoumi, Reihaneh Zarrizi, Julie Bonne Køhler, Sven Kjellström, Vito Alessandro Lasorsa, Mario Capasso, Haian Fu, Ramin Massoumi

**Affiliations:** 1 Department of Laboratory Medicine, Translational Cancer Research, Lund University, Medicon Village, Lund, Sweden; 2 Department of Pharmacology and Emory Chemical Biology Discovery Center, Emory University School of Medicine, Atlanta, USA; 3 Biotech Research and Innovation Centre, University of Copenhagen, 2200 Copenhagen, Denmark; 4 Department of Clinical Sciences Lund, Neurology, Lund University, Faculty of Medicine, Lund, Sweden; 5 Università degli Studi di Napoli Federico II, Dipartimento di Medicina Molecolare e Biotecnologie Mediche, via Sergio Pansini 5, 80131 Naples, Italy; 6 CEINGE Biotecnologie Avanzate, Via G Salvatore 486, 80145 Naples, Italy; 7 IRCCS SDN, Istituto di Ricerca Diagnostica e Nucleare, Via Gianturco 113, 80143 Naples, Italy

## Abstract

BRCA1-associated protein 1 (BAP1) is a nuclear deubiquitinating enzyme that is associated with multiprotein complexes that regulate key cellular pathways, including cell cycle, cellular differentiation, cell death, and the DNA damage response. In this study, we found that the reduced expression of BAP1 pro6motes the survival of neuroblastoma cells, and restoring the levels of BAP1 in these cells facilitated a delay in S and G2/M phase of the cell cycle, as well as cell apoptosis. The mechanism that BAP1 induces cell death is mediated via an interaction with 14-3-3 protein. The association between BAP1 and 14-3-3 protein releases the apoptotic inducer protein Bax from 14-3-3 and promotes cell death through the intrinsic apoptosis pathway. Xenograft studies confirmed that the expression of BAP1 reduces tumor growth and progression in vivo by lowering the levels of pro-survival factors such as Bcl-2, which in turn diminish the survival potential of the tumor cells. Patient data analyses confirmed the finding that the high-BAP1 mRNA expression correlates with a better clinical outcome. In summary, our study uncovers a new mechanism for BAP1 in the regulation of cell apoptosis in neuroblastoma cells.

## Introduction

Neuroblastoma originates from the sympathetic nervous system and is composed of undifferentiated and poorly differentiated neuroblasts arising from the different stages of the sympathoadrenal lineage of neural crest origin^1^. The patient age, N-Myc amplification, deletion of the chromosome, and metastatic spread are important factors with regard to treatment decision and patient prognosis. Although, N-Myc has important prognostic value, amplification is only observed in about 25% of neuroblastoma cases and other factors contributing to high-risk neuroblastoma are not known^2^. Surgery, radiotherapy, and intensive induction chemotherapy with autologous stem cell transplantation are commonly used as treatment therapy for neuroblastoma patients. In addition, terminal differentiation therapy and immunotherapy is used as a current standard therapy for high-risk neuroblastomas in order to eliminate residual tumor cells that are resistant after chemotherapy and stem cell transplantation^3–5^.

Tumorigenesis in neuroblastoma can be caused by the upregulation of cell survival signaling and the lack of cellular apoptosis. Therefore, understanding the mechanism that leads to cell survival pathways can provide avenues for the development of novel therapeutics. The hallmark of apoptosis is the activation of caspases that coordinate cleavage of substrates leading to cell death. Generally, apoptosis is divided into the extrinsic and intrinsic pathway. The extrinsic pathway is mediated via cell surface death receptors; whereas, the intrinsic pathway is mediated via the mitochondrial^6–9^. DNA-damaged cells are eliminated by the intrinsic pathway in which the Bcl-2 family of proteins plays a critical role^9^. This family is divided into anti-apoptotic proteins (including Bcl-2, Bcl-XL, and Mcl-1), and pro-apoptotic proteins, which is further divided into multi-domain proteins, such as Bax, Bak, and BH3-only proteins, including Bad, Bim, and HRK/DP5. Tumor cells often increase the expression of anti-apoptotic Bcl-2 members to avoid cancer cells undergoing apoptosis. Indeed, in a large subset of neuroblastoma patients, an elevated level of Bcl-2 has been detected^10,11^. Besides regulating cancer cell survival, chemotherapy-induced apoptosis is blocked in neuroblastoma through the involvement of the Bcl-2 protein family^12^.

BRCA1-associated protein 1 (BAP1) is a deubiquitinating enzyme that was discovered through its interaction with the RING finger domain of tumor suppressor protein BRCA1^13,14^. BAP1 is a tumor suppressor gene deleted or mutated in various human cancer types, including breast, lung, renal cell carcinoma, metastatic uveal melanomas, and malignant pleural mesotheliomas^13,15–18^. In mice, the disruption of BAP1 leads to the development of myeloid neoplasia^19^; whereas, the expression of BAP1 suppresses the growth of non-small cell lung carcinoma cells in nude mice^15^. Another function of BAP1 is to prevent abnormal mitotic spindle formation and genome instability via the deubiquitination of α-tubulin in human breast cancer cells^20^. BAP1 can also interact with several proteins associated with chromatin and transcription regulation, such as sex combs-like ASXL1 and ASXL2, forkhead transcription factors FOXK1 and FOXK2, lysine-specific demethylase 1B (KDM1B), O-linked N-acetylglucosamine transferase (OGT), and host cell factor 1 (HCF-1)^21–23^. Previous studies have shown that BAP1 has a role in cell cycle regulation and cell proliferation^15,19,22,24,25^. Further, BAP1 can regulate the cell cycle by influencing the expression of E2F1 target genes in uveal melanoma cells^26^. The regulation of DNA damage response by BAP1 is mediated via rapid poly(ADP-ribose)-dependent recruitment of the polycomb repressive deubiquitination (PR-DUB) complex to sites of DNA damage^27,28^. Phosphorylation of BAP1 at S592 is an important regulatory mechanism to dissociate BAP1 from chromatin and regulate-specific genes during DNA replication and repair^29^.

In this study, we investigated the role of BAP1 as a tumor suppressor gene in neuroblastoma based on the 3p-chromosomal location of BAP1 and that alteration in chromosome arms 3p is a common event in neuroblastoma. It was found that the pro-apoptotic function of BAP1 is mediated via binding to 14-3-3 protein, which further facilitated cell death signaling in neuroblastoma.

## Materials and methods

### Cell culture

The human neuroblastoma cell lines were cultured for 5 days at 37 °C and 5% CO_2_ as follows: IMR32 (ATCC, CCL-127), SK-N-SH-RA^30,31^, SK-N-FI (ATCC, CRL-2142), SK-N-SH (ATCC, HTB-11), and SK-N-DZ (ATCC, CRL-2149) cells were cultured in RPMI 1640 Medium (HyClone, Thermo Scientific, USA), supplemented with 10% FBS (Sigma-Aldrich, Sweden), and 0.1% penicillin/streptomycin (Gibco, Life Technologies, UK). SK-N-Be2c cells (ATCC, CRL-2271) were cultured in MEM (HyClone, Thermo Scientific, USA) supplemented with 10% FBS (Sigma-Aldrich) and 0.1% penicillin/streptomycin (Gibco).

### Animal model

SK-N-Be2c cells (5.0 × 10^6^ cells in 0.1 mL PBS) stably expressing BAP1 or control expression plasmid (10 in each group) and SK-N-FI cells were subcutaneously injected into the dorsal left or right flank of 4-week-old female NMRI-nu/NMRI-Foxn1^nu/nu^ (Janvier Labs). The animals were maintained under specific pathogen-free (SPF) conditions. All experimental procedures were approved by the Malmo and Lund Animal Ethics Committee with the ethical number M129-15. The tumor growth was followed by measuring the volume every fifth day. The subcutaneous tumors were collected, weighed, and chosen for further study.

### Transient transfection

Transient transfection assays were carried out in six-well plates at 60% confluence, using PolyFect Transfection Reagent (Qiagen) or Lipofectamine^®^2000 Reagent (Invitrogen, life technologies) in accordance with the manufacturer’s recommendations. Twenty-four hours prior to transfection, cells were seeded in medium containing 10% FBS. Cells were incubated at 37 °C and 5% CO_2_ for 24–48 h. The medium was removed and the cells washed with PBS, after which fresh serum-containing medium was added to the cells. DNA, 1.5–4.0 µg, was mixed with an appropriate volume of OptiMEM (Sigma), followed by the addition of 10–25 µl PolyFect transfection reagent. The samples were incubated at room temperature for 10 min, in order to allow the complex formation to be completed, prior to being transferred to the cells. Cells were subsequently incubated for 24–48 h, collected and used for further analyses.

Transient transfection of siRNA (10 µM) was performed in six-well plates at 60% confluence, using Lipofectamine^®^ RNAiMAX Reagent (Invitrogen, life technologies) following the manufacturer’s instructions. Forty-eight hours post transfection, cells were collected and used for further analyses. The siRNA sequences were listed as following:

14-3-3 zeta: 5′-AAAGTTCTTGATCCCCAATGC-3′

Crtl. GFP: 5′-GACCCGCGCCGAGGTGAAGGTTT-3′

### Drug treatment

The non-transfected SK-N-RA cells in six-well plates were treated for 24 h with BV02 (SML0140, Sigma-Aldrich) at different concentrations (5, 20, and 100 µM). The percentages of apoptotic cells were determined using the Burkers chamber.

### Cell lysis and subcellular fractionation

Cells were grown on 10 cm plates to 80% confluence, collected in PBS, and lysed in buffer comprising 1 M Tris-HCl (pH 7.6), 5 M NaCl, 0.5 M EDTA, 1% Triton X-100, and Complete® protease inhibitor cocktail (Roche, Germany). The extraction of protein from tumor tissue was prepared after samples were homogenized and sonicated in cold RIPA buffer (50 mM Tris-HCl, pH 7.4, 150 mM NaCl, 1.0 mM EDTA, 0.1% SDS, 1.0% Triton X-100, 1.0% sodium deoxycholate) freshly supplemented with protease inhibitor cocktail (Roche, Germany). After centrifugation at 10,000×*g* for 15 min at 4 °C, the protein in the supernatant was measured using the Protein concentration and was assessed using the Bradford method and sample concentrations were adjusted accordingly. Lysates were boiled for 5 minutes at 100 °C with a 2× sample buffer, containing 1 mM dithiothreitol (DTT).

For cell fractionation, cells were first resuspended in ice-cold buffer A (10 mM HEPES (pH 7.9), 10 mM KCl, 1.5 mM MgCl_2_, 0.34 M sucrose, 10% glycerol, 1 mM DTT, complete protease inhibitors EDTA-free (Roche, Germany) and 0.1 mM phenylmethylsulfonyl fluoride), 0.1% Triton X-100 was added followed by incubation for 10 min on ice, and centrifuged at 4 °C for 5 min at 1300×*g*. The supernatant containing cytosolic fraction was removed and the pellet was enriched in chromatin and washed once in buffer A before re-suspension in protein sample buffer (2% SDS, 312.5 mM Tris, pH 6.8, 5% glycerol, 0.003% BFB, and 50 mM DTT) and boiled in sample buffer for 10 min and analyzed by western blotting using anti-tubulin (Sigma-Aldrich, Sweden) and anti-LaminB (Thermo Scientific, USA) as molecular markers for the cytosolic and nuclear fractions, respectively.

### Cell cycle synchronization

Cells were synchronized in G0/G1 or G2/M phase either by serum starvation for 48 h or nocodazole treatment for 18 h (100 ng/ml, sigma) respectively. The synchronized cells were released from their respective arrested phase by the addition of 10% serum containing RPMI (HyClone, Thermo scientific, USA), and samples were taken at various times. The cells were fixed by dripping ice-cold 70% ethanol while vortexing the cells vigorously and stored for 12–24 h at −20 °C. Ethanol-suspended cells were centrifuged and the ethanol carefully decanted. The cells were washed once with PBS and then resuspended with NucleoCounter Solution 3 (1 μg/ml 4′,6-diamidino-2-phenylindole, 0.1% Triton X-100 in PBS), followed by incubation for 5 min at 37 °C. Samples of 10 μl volume were loaded into a slide chamber (NC-slide A8), and the DNA fragmentation protocol was used according to the manufacturer’s instructions (ChemoMetec). To assess the cell cycle profile following nocodazole synchronization, ethanol-fixed cells were washed twice with PBS, resuspended in propidium iodide (PI) staining solution (20 μg/ml PI, 200 μg/ml Rnase A in PBS containing 0.1% Triton X-100), and incubated at 37 °C for 30 min. For each condition, 10,000 cells were analyzed by a flow cytometer (BD FACS Verse^™^) and the cell cycle profile was analyzed using the FlowJo v10 software.

### Western blot analysis

Western blotting was carried out, following standard protocol. The membrane was incubated with primary antibody in 5% BSA followed incubation with corresponding horseradish peroxidase (HRP)–conjugated secondary antibody (DAKO, Denmark) for 1 h at RT. Bands were detected using immunochemical detection according to the manufacturer’s instruction (Santa Cruz, USA). For the chemiluminescent reaction, Supersignal Substrate (Thermo Scientific) was used according to manufacturer’s instructions. Chemiluminescence was detected with an LAS-1000 charge-coupled device camera (Fujifilm) and images were processed using the ImageJ software.

### Quantitative PCR

The cells were rinsed in cold PBS and total RNA extracted using the Perfect Pure RNA tissue kit (5 PRIME, Germany) according to manufacturer’s instruction. The purity of RNA was analyzed and quantified by a NanoDrop spectrophotometer (Saveen Werner, Sweden) and used for cDNA synthesis according to the manufacturer’s instruction (QPCR cDNA synthesis kit, Stratagene, USA). PCR runs were performed in the QuantStudioTM 7 Flex System using SYBR®Green Reagent (Applied Biosystems), with the following program; 2 min 50 °C, 10 min in 95 °C followed by 40 three-step cycles consisting of 95 °C for 20 s and 60 °C for 30 s and 72 °C for 1 min. The following primers were used:

BAP1_S: 5′-GACCCAGGCCTCTTCACC-3′

BAP1_A: 5′-AGTCCTTCATGCGACTCAGG-3′

14-3-3 Zeta_S: 5′-CCTGCATGAAGTCTGTAACTGAG-3′

14-3-3 Zeta_A: 5′-GACCTACGGGCTCCTACAACA-3′

GAPDH_S: 5′-TGCACCACCAACTGCTTAGC-3′

GAPDH_A: 5′-GGCATGGACTGTGGTCATGAG-3′

### Retrovirus production and transduction

Retroviral vector alone or vector encoding wild-type FLAG and HA-tagged BAP1 (plasmid 22539) containing a puromycin resistance gene for selection of stably transduced cells were obtained from Add gene. Retrovirus production was performed by Research engineer, Vector Unit at Lund University, Sweden. Cells were seeded in six-well plates with 40% confluency in RPMI medium (HyClone, Thermo-scientific, USA) supplemented with 10% FBS, 2 mM l-glutamine, 100 units/mL penicillin and 100 µg/mL streptomycin (Sigma-Aldrich, Sweden). Next day, the medium was changed with 1 ml medium containing hexadimethrine bromide (Sigma-Aldrich, Sweden; final concentration 8 mg/ml) to each well. A total of 10 µl of retroviral particles was added to appropriate wells and cells were incubated 24 h at 37 °C in a humidified incubator in an atmosphere of 5–7% CO_2_. The medium was changed with 1 ml RPMI containing puromycin (Sigma-Aldrich, Sweden). The transduction efficiency was initially evaluated using western blot and Immunofluorescence staining.

### Apoptosis and cell proliferation

Cells were grown on six-well plates, collected at 24 or 48 h post transfection, and counted using the Burkers chamber, or Countess® Automated Cell Counter (Invitrogen). For apoptosis analyses, the cells were fixed in PFA on coverslips, and stained with a Vindelöv solution, containing propidium iodide. After washing, the coverslips were mounted onto glass slides and examined by fluorescence microscopy. Cells were scored for apoptosis, based on nuclear morphology. Apoptosis was furthermore evaluated, using NucleoCounter NC-3000 (Chemometec), in conformity with the DNA fragmentation assay. Briefly, transfected and non-transfected cells (controls) were grown on 6-well plates. The cells were collected by trypsinization and pooled with the cells floating in the medium. After a short centrifugation, the supernatant was removed and the precipitated cells were washed once with PBS. After a second centrifugation, the cells were resuspended in a small volume of PBS, and the single-cell suspensions were added to 70% ethanol for fixation. The samples were vortexed and stored for 12–24 h at −20 °C. The ethanol-suspended cells were centrifuged and the ethanol carefully decanted. Cells were washed once with PBS and then resuspended with NucleoCounter Solution 3 (1 µg/ml DAPI, 0.1% Triton X-100 in PBS), followed by incubation for 5 min at 37 °C. The samples of 10 µl volume were loaded into a slide chamber (NC-slide A8), and the DNA Fragmentation protocol was employed according to manufacturer’s instructions (Chemometec).

EdU incorporation: A thymidine analog, 5-ethynyl-2′-deoxyuridine (EdU), incorporation was used to measure cell proliferation using Click-iT® EdU Alexa Flour 488 cell proliferation assay kit (Thermo Fisher Scientific). Briefly, BAP1-low and BAP1-overexpressing SK-N-SH cells were seeded in a six-well plate (250 × 10^3^ cells/well) in DMEM with 10% FBS containing EdU (5 µM/well) and incubated at 37 °C. After 24, 48, and 72 h, the cells were collected, washed twice in PBS containing 1% BSA and click-iT reaction was performed on fixed cells before FACS analysis was carried out to determine the fraction of proliferating cells. To define EdU labeled cell fractions (Click-iT EdU Alexa Flour 488 positive), EdU-unlabeled SK-N-SH cells were used as a negative control after simultaneously stained with click-iT reaction solution. For each condition, 20,000 events were acquired by a flow cytometer (BD FACS Verse™) and the percentage indicated in the bar graph was analyzed using FlowJo v10 software. The results are the mean of two independent experiments.

### Annexin V-phycoerythrin staining and fluorescence-activated cell sorting analysis

Cells were stained using Annexin V-phycoerythrin (PE) apoptosis detection kit (BD Pharmingen) according to the manufacturer’s recommendations. Briefly, the floating cells in the medium together with the respective adhered cells were collected and washed twice with ice-cold PBS before suspended in 100 μl binding buffer (1 × 10^6^/ml). Next, Annexin V-PE (5 µl) and 7-AAD (5 µl) were added and the mixture was incubated for 15 min in the dark. Finally, 400 µl binding buffer was added to the cells and analyzed using a flow cytometer (BD FACS Verse^™^). The data analysis was performed using FlowJo v10 software.

### Measurement of mitochondrial membrane potential

The mitochondrial membrane potential (ΔΨm) was evaluated by flow cytometric analysis with JC-1 staining. Stably transduced neuroblastoma cells both with empty vector (control) and BAP1 were collected after trypsinization. For arsenic induction, cells were seeded in 100 mm Petri dish (5 × 105 cells) for 48 h, and then treated with arsenic trioxide (6 µM) and collected after 24 h. The cells resuspended in PBS (1 × 10^6^) following washing steps were incubated with 5 µM JC-1 at 37 °C, 5% CO_2_, for 30 min in the dark. For each condition, 10,000 events were acquired by a flow cytometer (BD FACS Verse™) and the percentage indicated in the dot plot was analyzed using FlowJo v10 software.

### LC–MS/MS analysis

MS analyses were carried out on an Orbitrap Fusion Tribrid MS system (Thermo Scientific) equipped with a Proxeon Easy-nLC 1000 (Thermo Fisher). Injected peptides were trapped on an Acclaim PepMap C18 column (3 µm particle size, 75 µm inner diameter × 20 mm length). After trapping, gradient elution of peptides was performed on an Acclaim PepMap C18 100 Å 3 μm, 150 mm, 75 μm). The outlet of the analytical column was coupled directly to the mass spectrometer using a Proxeon nanospray source. The mobile phases for LC separation were 0.1% (v/v) formic acid in LC–MS grade water (solvent A) and 0.1% (v/v) formic acid in acetonitrile (solvent B). Peptides were first loaded with a constant pressure mode with a flowrate of solvent A onto the trapping column. Subsequently, peptides were eluted via the analytical column at a constant flow of 300 nl/min. During the elution step, the percentage of solvent B increased in a linear fashion from 5 to 10% in 2 min, then increased to 25% in 85 min and finally to 60% in a further 20 min. The peptides were introduced into the mass spectrometer via a Stainless steel emitter 40 mm (Thermo Fisher) and a spray voltage of 2.0 kV was applied. The capillary temperature was set at 275 °C. Data acquisition was carried out using a data-dependent top N method with cycle time of 2 s. The master scan was performed in the Orbitrap in the range of 380–1580 m/z at a resolution of 60,000 FWHM. The filling time was set at maximum of 50 ms with limitation of 4 × 105 ions. Ion trap CID-MS2 was acquired using parallel mode, filling time 50 ms with limitation of 1.5 × 104 ions, a precursor ion isolation width of 0.7 m/z and resolution of 30,000 FWHM. Normalized collision energy was set to 35%. Only multiply charged (2+ to 4+) precursor ions were selected for MS2. The dynamic exclusion list was set to 30 s and a relative mass window of 5 p.p.m.

### Pull-down assay

Ni-NTA pull-down assay: SK-N-RA cells were transfected with His6-ubiquitin and/or BAP1 expressing plasmids. Cells were lysed after 24 h in 8 M UREA, 100 mM Na_2_HPO_4_/NaH_2_PO_4_; 10 mM Tris, pH 8.0, 10 mM imidazole and 5 mM DTT, and lysates (1 mg) were incubated with 40 μl Ni-NTA Superflow (Qiagen) overnight at room temperature. The resin was washed three times with lysis buffer, once with 8 M UREA, 100 mM Na_2_HPO_4_/NaH_2_PO_4_; 10 mM Tris, pH 6.3; 10 mM imidazole; 5 mM DTT and once more with lysis buffer before eluting proteins with lysis buffer containing 200 mM imidazole. Isolated proteins were subjected to immunoblotting using antibodies against ubiquitin at 1:1000 (SC-8017, Santa Cruz) or pan-14-3-3 at 1:500 (SC-629, Santa Cruz).

GST pull-down assay: Co-transfected cells were lysed in 1% NP-40 buffer (20 mM Tris (pH 8.0), 137 mM NaCl, 5% glycerol, 1% nonident P-40, Protease Inhibitor (Sigma, P8340, USA) and phosphatase inhibitor (Sigma, P5726, USA) and centrifuged for 10 min at 4 °C. Aliquots were taken for input control and the remaining lysate were incubated with pre-washed (with lysis buffer) glutathione-conjugated sepharose beads (GE, 17075605, Sweden) for 3 h at 4 °C. After washing three times with 1% NP-40 buffer, the beads were resuspended in sample buffer (Bio-Rad, 1610737, USA) and boiled at 95 °C for 5 min. Then the eluted samples and whole-cell lysate were subjected to sodium dodecyl sulfate-polyacrylamide gel electrophoresis (SDS-PAGE) and analyzed by western blot with the anti-Flag antibody at 1:3000 (Sigma, A8592, USA), anti-GST antibody at 1:3000 (Sigma, A7340, USA) and anti-β-Actin antibody at 1:5000 (Sigma, A5441, USA).

### Time-resolved fluorescence resonance energy transfer (TR-FRET) assay

The TR-FRET assay was carried out with purified proteins in 384-well black solid bottom plates (Corning, 3573, USA). His-BAP1 protein (Boston Biochem, E-345, USA), control GST protein (Abcam, ab70456, USA) and purified GST-14-3-3 proteins were mixed in a total volume of 30 µl FRET buffer (20 mM Tris (pH 7.0), 50 mM NaCl, 0.01% NP-40). Anti-GST-Terbium antibody (Cisbio, 61GSTTLB, USA) and anti-His-D2 antibody (Cisbio, 61HISDLA, USA) were added to each well at the final dilution of 1:1000 and 1:500, respectively. The FRET signals were detected using Envision spectrophotometer (Laser excitation at 337 nm, emission for the donor at 620 nm and emission for acceptor at 665 nm). The data were presented as the ratio of (counts at 665/counts at 620 nm) × 10^4^ with standard deviation calculated from triplicate samples.

### Immunofluorescence and confocal microscopy

Cells were cultured on coverslips in six-well plates for 24 h and then fixed in paraformaldehyde (PFA) (4% for 15 min). Next, the cells were permeabilized with 0.3% Triton X-100 solution, after which they were blocked with 1% BSA and 5% goat serum for 30 min to prevent non-specific antibody binding, and subsequently incubated for 1 h with primary antibodies against BAP1 (Clone C-4; sc-28383, Santa Cruz, USA), α-tubulin (Sigma-Aldrich, Sweden), and FLAG (Sigma-Aldrich, Sweden). After washing coverslips, fluorescent antibodies (Alexa 488 goat anti-mouse or Alexa 546 goat anti-rabbit (Invitrogen, USA)) were applied, then the coverslips were washed and mounted in VECTASHIELD® with diamidino-2-phenylindole (DAPI) (Vector Laboratories). The images were obtained using a Zeiss LSM710 confocal microscope.

### RT^2^ profiler PCR array analysis

The apoptosis pathway-focused gene expression profiling was performed using a 384-well human RT^2^ Profiler PCR Array PAHS-012ZE (Qiagen). Following the total RNA extraction, cDNA was synthesized from SK-N-RA cells transiently transfected with an empty vector used as control (GFP) and full-length BAP1 based on the manufacturer’s instructions. In this array, 84 different genes involved in programmed cell death were analyzed based on SYBR Green real-time PCR using the QuantStudio™ 7 Flex (Applied Biosystems™). Normalization was performed using the five different housekeeping genes included in the array and the fold-change was calculated using the RT^2^ Profiler PCR Array Data Analysis.

### Gene expression data for survival

BAP1 and Bax normalized gene expression array data (Affymetrix HG-U133A [GPL96] and HG-U133B [GPL97] platforms; GSE3446) of 102 neuroblastoma patients were downloaded from the website R2: Genomics Analysis and Visualization Platform (http://r2.amc.nl). To test the association of gene expression levels with relapse-free survival, individual gene expression profiles were dichotomized by median split into “high” or “low” expression groups, and Kaplan–Meier survival curves were plotted for each group.

### Statistical analyses

Statistical analyses were performed using SigmaPlot software. The results are expressed as mean ± s.e.m. or as a percent. *P*-values <0.05 were deemed statistically significant. Statistical comparisons were assessed by ANOVA or by Student’s *t*-test (*p* < 0.05).

## Results

### Reduced levels of BAP1 in neuroblastoma cells

**Fig. 1:**
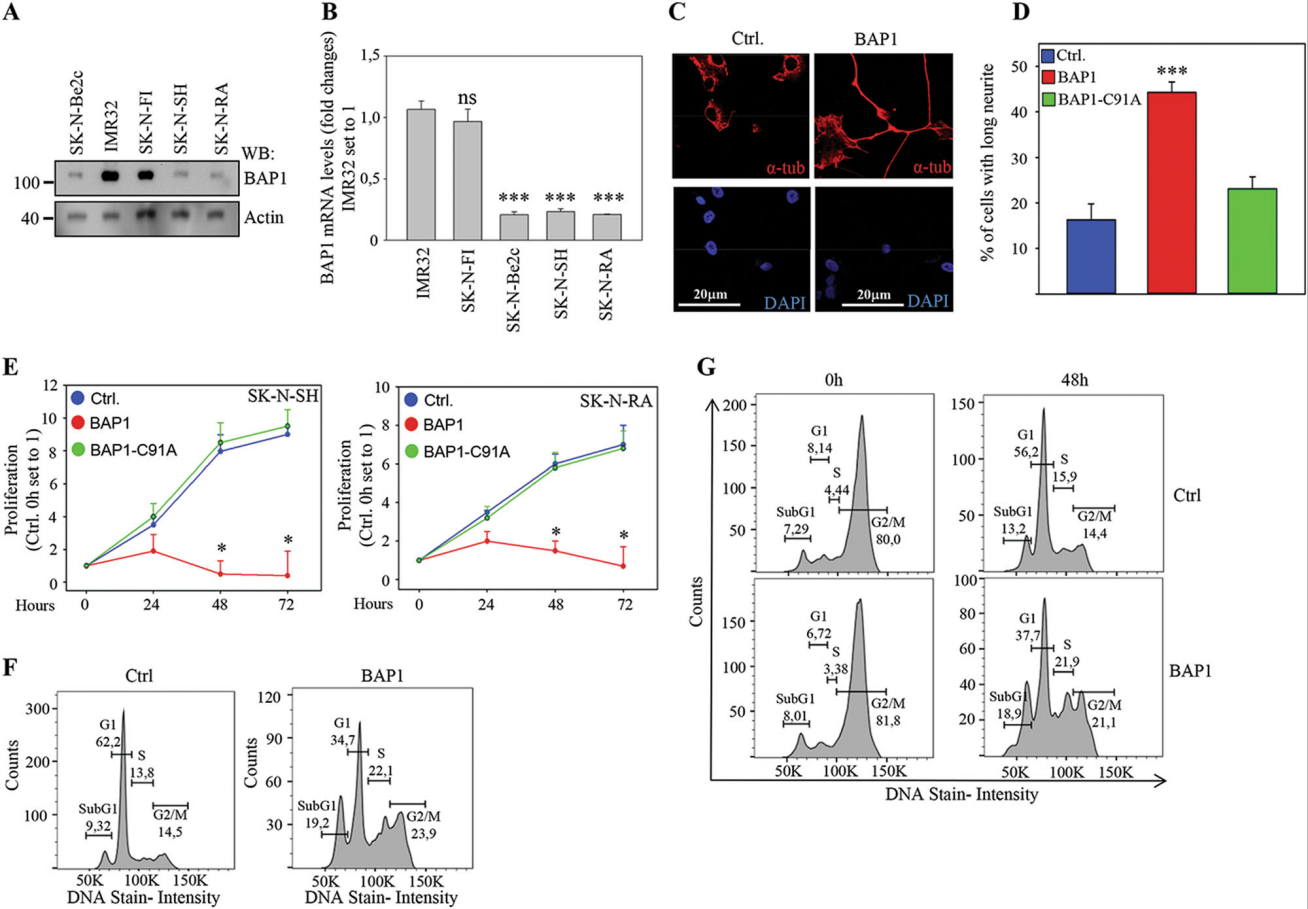
Reduced expression of BAP1 in neuroblastoma cell lines. **a** Western blot analysis of BAP1 and actin expression in SK-N-Be2c, IMR32, SK-N-F1, SK-N-SH, and SK-N-RA cells (*n* = 5). **b** Fold changes in BAP1 mRNA expression, measured using real-time reverse transcription PCR (qRT-PCR) of cDNA from SK-N-Be2c, IMR32, SK-N-F1, SK-N-SH, and SK-N-RA cells. The IMR32 mRNA expression is set to 1 (mean ± s.e.m., *n* = 3, ***P < 0.001). **c** Phase-contrast microscopy and confocal images of SK-N-BE2c cells transiently transfected with empty plasmid (Ctrl.) or BAP1 expression plasmid for 72 h. The neurite outgrowth in BAP1 expressing cells is shown by staining with anti-α-tubulin antibody and DAPI stain nuclei (blue) (*n* = 5). **d** The morphological effects of the overexpression of BAP1 or empty plasmid (Ctrl.) in SK-N-BE2c cells by counting the number of cells with cell processes longer than the length of two cell bodies (mean ± s.e.m., *n* = 3, ***P < 0.001). **e** The cell proliferation was determined by cell counting after 24–96 h using lentivirus infected SK-N-SH (upper panel) and SK-N-RA (lower panel) with GFP (Ctrl.) or BAP1. The experiment was performed in triplicate. Data are presented as mean ± s.e.m. (*n* = 4, *P < 0.05). **f**, **g** Representative histogram plots (*n* = 3) displaying changes in cell cycle distribution as a result of BAP1 overexpression assessed using propidium iodide (PI) staining in SK-N-Be2c cells unsynchronized (**f**) or synchronized by nocodazole (100 ng/ml) for 18 h (**g**). The synchronized cells were released after 48 h and cell cycle profile experiment was performed using BD FACS Verse™. The histogram plot and the percentage of cells in sub-G1, G1, S, and G2/M were determined using FlowJo v10 software (TreeStar)

In order to investigate whether BAP1 expression is altered in neuroblastoma, we studied the levels of BAP1 expression in different neuroblastoma cell lines. It was found that BAP1 was significantly downregulated at the mRNA and protein levels in the SK-N-BE2c, SK-N-SH, SK-N-RA, KcN-69n, and SH-S454 cells compared to IMR32 and SK-N-F1 cells (Fig. 1a, b and Supplementary Fig. 1). To investigate the role of BAP1 in neuroblastoma, SK-N-SH, SK-N-RA, and SK-N-BE2c cells were virally infected with full-length BAP1 tagged with HA and FLAG (Supplementary Fig. 2). Monitoring cell morphology of neuroblastoma cells expressing full-length BAP1 showed that these cells but not the cells expressing a control plasmid or the cells expressing a catalytically inactive mutant of BAP1 (BAP1-C91A) facilitated neurite outgrowth 72–96 h post transfection (Fig. 1c, d and Supplementary Fig. 3). Neurite outgrowth can indicate the non-proliferative stage of the cells and this can interfere with the survival of neuroblastoma cells^32^. To determine whether BAP1 can affect cell growth, we analyzed the proliferation rate of BAP1, BAP1-C91A, and GFP control (Ctrl.) expression plasmid lentivirus infected cells. Rescue expression of BAP1, but not BAP1-C91A in two neuroblastoma cells lines reduced the proliferation rate at 48 and 72 h, compared with control cells (Fig. 1e and supplementary Fig. 4). The effect of BAP1 overexpression in altering cell cycle progression was demonstrated in non-synchronized SK-N-Be2c cells where the frequency of cells in the S, G2/M, and subG1 phases were higher compared to the control or BAP1-C91A expressing cells (Fig. 1f and Supplementary Fig. 5). To confirm this result, we synchronized the cells in G2/M phase by treating the cells with nocodazole (Fig. 1g, left panels). The release of synchronization after 48 h showed an accumulation of cells in the S, G2/M, and subG1 phases in BAP1 expressing cells compared to control cells (Fig. 1g, right panels).

### BAP1 promotes cell death in neuroblastoma cells

**Fig. 2:**
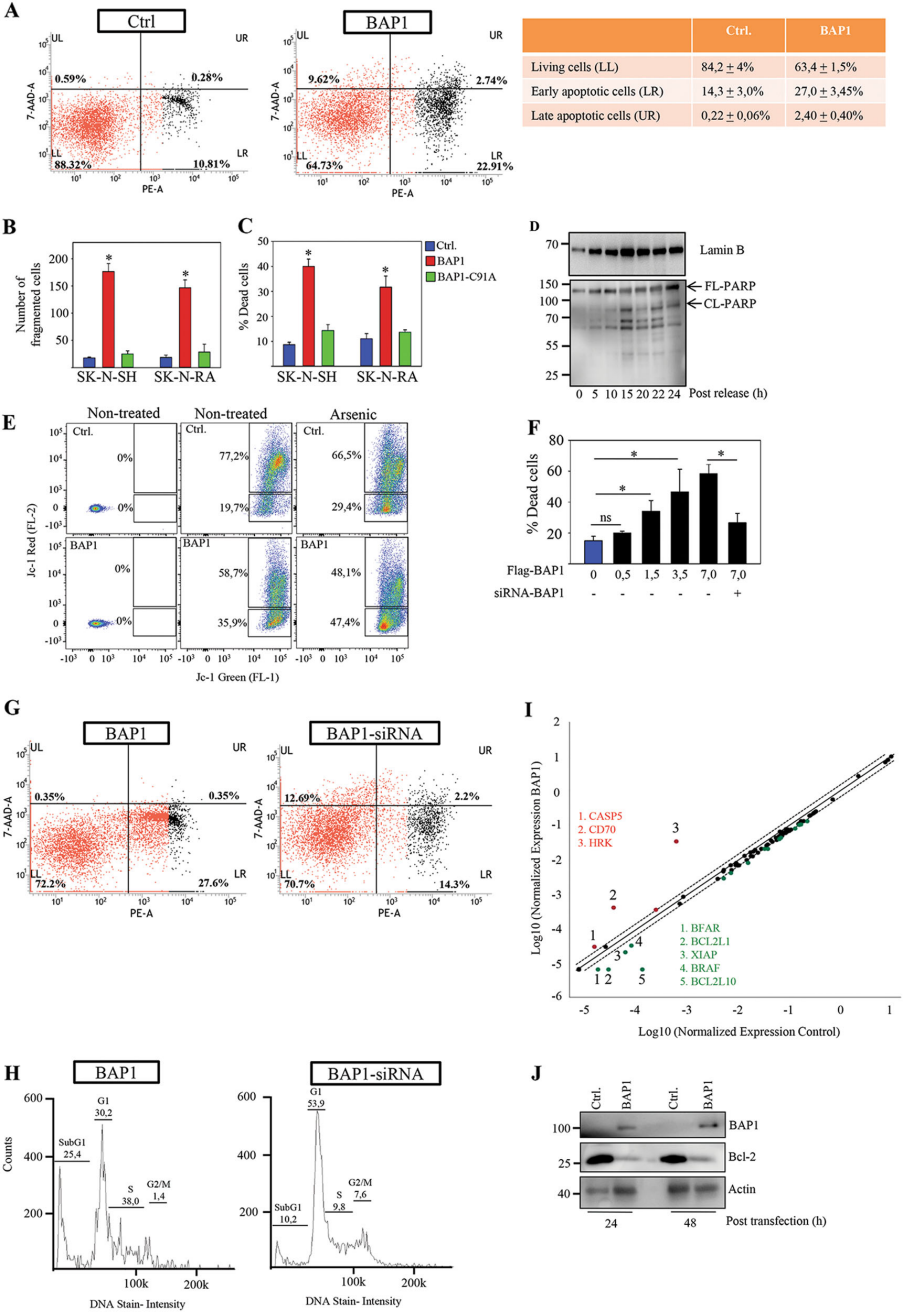
BAP1 induces cell death and downregulates expression of survival genes. **a** SK-N-SH cells were transiently transfected with control or BAP1 expression plasmid for 48 h and analyzed for apoptosis by flow cytometry following PE and 7-AAD staining. Quadrants are as follows: lower left: viable cells; lower right: early apoptotic; upper right: late apoptotic/necrotic. The numbers in quadrants represent the percentage of the viable cells (lower left), early apoptotic (lower right), and late apoptotic/necrotic (upper right). The table (right panel) shows the distribution of viable cells, early apoptotic and late apoptotic/necrotic from three independent experiments. **b** SK-N-RA or SK-N-SH cells were transfected with mock or FLAG-BAP1 for 24 h. Cells were fixed and treated for DNA fragmentation assay. Data represent the percentage of cells with fragmented DNA. Data are presented as mean ± s.e.m. (*n* = 3, *P < 0.05). **c** Cell death visualized by propidium iodide (PI) staining in SK-N-RA and SK-N-SH cells infected with control (GFP) or FLAG-tagged full-length BAP1. Results are mean ± s.e.m. of percentage cells (*P < 0.05) with PI staining from three independent experiments counting 200 cells in triplicate. **d** Extracts from synchronized and released SK-N-RA cells transfected with FLAG-tagged full-length BAP1 were examined by western blotting (WB) in relation to post-release time points with an anti-PARP and anti-laminB. Arrowheads indicate FL-PARP: full-length PARP and CL-PARP: cleaved PARP. (*n* = 3). **e** Effect of BAP1 overexpression in altering mitochondrial membrane potential in SK-N-Be2c cells was measured by flow cytometry following JC-1 staining. Cells without JC-1 staining were used to demarcate the gates prior to measure the ratio of red (mitochondria with a non-depolarized ΔΨ) and green (depolarized ΔΨ) fluorescence emissions after JC-1 staining (5 μM). Mitochondrial membrane potential was shown to be altered in BAP1-overexpressing cells alone (35.9%) or following arsenic treatment (6 μM, 47.4%) (*n* = 3). **f** SK-N-BE2c cells were non-transfected (0 µg/µl) or transiently transfected with different concentration of full-length FLAG-tagged BAP1 expression plasmid (0.5 µg/µl, 1.5 µg/µl, 3.5 µg/µl, and 7.0 µg/µl) for 48 h and transfected with siRNA oligos against BAP1 for another 48 h as indicated in the figure. Results are mean ± s.e.m. of percentage cells (*P < 0.05) with PI staining from three independent experiments counting 200 cells in triplicate. **g** SK-N-SH cells were transiently transfected with full-length Flag-tagged BAP1 expression plasmid (7.0 µg/µl) for 48 h and transfected with siRNA oligos against BAP1 for 48 h. The cells were treated with Annexin V-PE and 7-AAD before flow cytometry analysis. Representative experiment shows numbers in quadrants represent the percentage of the viable cells (lower left), early apoptotic (lower right), and late apoptotic/necrotic (upper right) (*n* = 3). **h** SK-N-SH cells were transiently transfected with full-length FLAG-tagged BAP1 expression plasmid (7.0 µg/µl) for 48 h and transfected with siRNA oligo against BAP1 for 48 h. Cells were fixed and treated with DAPI. Representative experiment shows alterations in DNA content and cell cycle profiles using Nucleocounter NC-3000 (*n* = 3). **i** The Human Apoptosis RT² Profiler PCR Array revealed up- and downregulated genes involved in programmed cell death in SK-N-RA cells transiently transfected with full-length FLAG-tagged BAP1 compared to control (empty vector). The level of relative expression for each particular gene in the two samples are plotted in log–log scatter plot after normalization against five housekeeping genes. The line in the middle indicates relative fold changes. Red color indicates upregulated and green color indicates downregulated genes (*n* = 2). **j** Western blot analysis of full-length BAP1 expression in SK-N-RA cells analyzed by blotting with an anti-Bcl-2, anti-BAP1, or anti-actin antibody (*n* = 3)

Next, we investigated whether the accumulation of BAP1 expressing cells in subG1indicates cell death. The percentage of Annexin V positive cells (Fig. 2a) or cells having fragmented DNA (Fig. 2b) were significantly higher in the BAP1 expressing cells compared to BAP1-C91A or control cells (Fig. 2b). PI staining also displayed a higher number of BAP1 expressing cells undergoing cell death compared to BAP1-C91A or control cells (Fig. 2c). In cell cycle synchronized neuroblastoma cells, we monitored the commitment of BAP1 expressing cells to apoptosis after release. Poly (ADP-ribose) polymerase (PARP) cleavage was detected within 15–22 h post release in BAP1 but not in control expressing cells (Fig. 2d and Supplementary Fig. 6). Investigating the mitochondrial membrane potential (ΔΨm) in non-treated or arsenic-treated cells showed an elevated number of JC-1 dye positive cells (Fig. 2e, middle and right panels) and an intense release of Mito Tracker into the cytosol (Supplementary Fig. 7) in BAP1 expressing cells compared to control cells. These results suggest that BAP1 interfere with the intrinsic apoptosis pathway in neuroblastoma cells. Furthermore, transient transfection of neuroblastoma with increasing concentrations of the BAP1-cDNA showed a direct correlation between levels of BAP1 expression and cell death (Fig. 2f and Supplementary Fig. 8). The comparison of growth rate between neuroblastoma cells revealed that low-expressing BAP1 cells, SK-N-DZ cells (Supplementary Fig. 9A) showed an elevated proliferation rate and cell cycle progression, as well as reduced cell death compared with BAP1 expressing SK-N-F1 cells (Supplementary Fig. 9B-D). In addition, low-expressing BAP1 cells, SK-N-Be2c cells develop tumors (90% of animals) in xenograft experiments compared to BAP1 expressing SK-N-FI cells (10% of animals) (Supplementary Fig. 9E). Further, downregulation of BAP1 in SK-N-FI cells by using small interfering RNA (siRNA) (Supplementary Fig. 10A) enhanced cell survival compared to siRNA treated control cells (Supplementary Fig. 10B-C). Treatment of BAP1-overexpressing cells with siRNA oligos against BAP1 reversed this effect and promoted survival of the cells (Fig. 2g), as well as reduced the subG1 population of the cells (Fig. 2h).

To identify the global BAP-mediated pro- and anti-apoptotic genes in neuroblastoma cells, we tested the expression profiles of 84 well-known genes involved in apoptosis and survival pathways. While few genes encoding pro-apoptotic proteins (HRK, CD70) were upregulated, other genes encoding anti-apoptotic proteins (BCL2L1, XIAP, BCL2L10, and BFAR) were downregulated in BAP1 expressing cells compared to the control cells (Fig. 2i). This result was confirmed by showing that the transient overexpression of BAP1 downregulates the total levels of Bcl-2, 24–48 h post transfection (Fig. 2j).

### BAP1 binding to 14-3-3 facilitate the apoptotic signaling in neuroblastoma

**Fig. 3:**
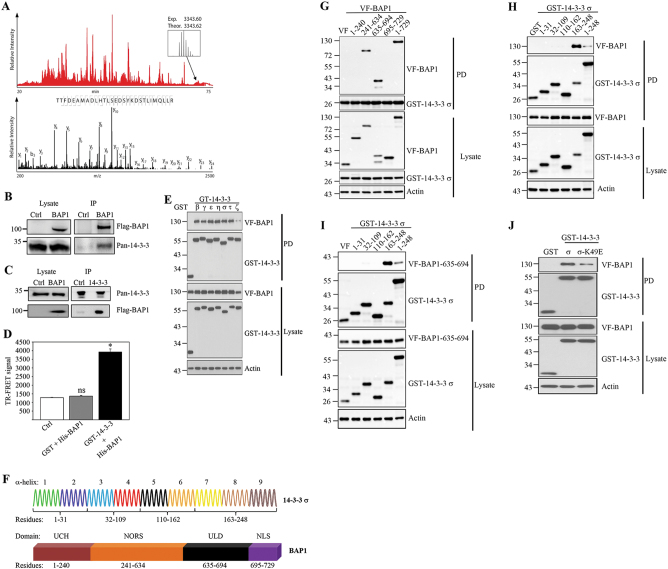
Identification of 14-3-3 protein as a novel binding partner to BAP1. **a** Representative LC–MS/MS chromatogram of in-gel digested sample depicted in red, an example of the peptide from 1433S protein eluted at 70.8 min. The Orbitrap mass spectra of the quadruply charged 1433S peptide sequence 191–224 (m/z 836.9045) enabled mass determination of the peptide (isotopic distribution depicted in gray). The assigned fragmentation pattern of sequence 191–224 peptide is given in black. **b**, **c** SK-N-RA cells were transfected with FLAG-tagged full-length BAP1 or Flag-empty vector (Ctrl) and lysates were immunoprecipitated with antibodies against FLAG (**b**) or 14-3-3 (**c**) and probed with FLAG to detect BAP1 or 14-3-3 as indicated in the figure (*n* = 3). **d** TR-FRET assay using FRET buffer (Ctrl), purified control GST (negative control), GST-14-3-3 protein, and His-BAP1 mixed in FRET buffer, followed by addition of anti-GST-Terbium and anti-His-D2 antibodies. The FRET signals were detected using an Envision spectrophotometer. The data were presented as the ratio of counts at 665/counts at 620 nm × 10^4^ with standard deviation calculated from triplicate samples (mean ± s.e.m., *n* = 3, *P < 0.05). **e** GST pull-down experiment using co-transfection of cells with GST-tagged 14-3-3 isoforms and full-length BAP1 followed by addition of GST-conjugated sepharose beads. The eluted whole-cell lysate was subjected to SDS-PAGE and analyzed by western blot using anti- FLAG, anti-GST, or anti-actin antibodies (*n* = 3). **f** Schematic representation of 14-3-3σ (9 alpha-helix) and BAP1 domains consisting of UCH (ubiquitin C-terminal hydrolases), NORS (non-regular secondary structure), ULD (ubiquitin like domain), and NLS (nuclear localization sequence). Residues numbers refer to amino acid positions. **g** GST pull-down experiment using co-transfection of cells with GST-tagged 14-3-3σ and FLAG-tagged full-length or deletion mutants of BAP1 followed by addition of GST-conjugated sepharose beads. The eluted whole-cell lysate was subjected to SDS-PAGE and analyzed by western blot using anti-FLAG, anti-GST, or anti-actin antibodies (*n* = 3). **h** GST pull-down experiment using co-transfection of cells with GST-tagged full-length or deletion mutant of 14-3-3σ and FLAG-tagged full-length BAP1 followed by addition of GST-conjugated sepharose beads. The eluted whole-cell lysate was subjected to SDS-PAGE and analyzed by western blot using anti-FLAG, anti-GST, or anti-actin antibodies (*n* = 3). **i** GST pull-down experiment using co-transfection of cells with GST-tagged full-length or deletion mutant of 14-3-3σ and full-length or FLAG-tagged deletion mutant of BAP1 (aa 635–694) followed by addition of GST-conjugated sepharose beads. The eluted whole-cell lysate was subjected to SDS-PAGE and analyzed by western blot using anti-FLAG, anti-GST, or anti-actin antibodies (*n* = 3). **j** GST pull-down experiment using co-transfection of cells with GST-tagged 14-3-3σ, 14-3-3σ mutant (σ-K49E), and full-length BAP1 followed by addition of GST-conjugated sepharose beads. The eluted whole-cell lysate was subjected to SDS-PAGE and analyzed by western blot using anti-FLAG, anti-GST, or anti-actin antibodies (*n* = 3)

**Fig. 4:**
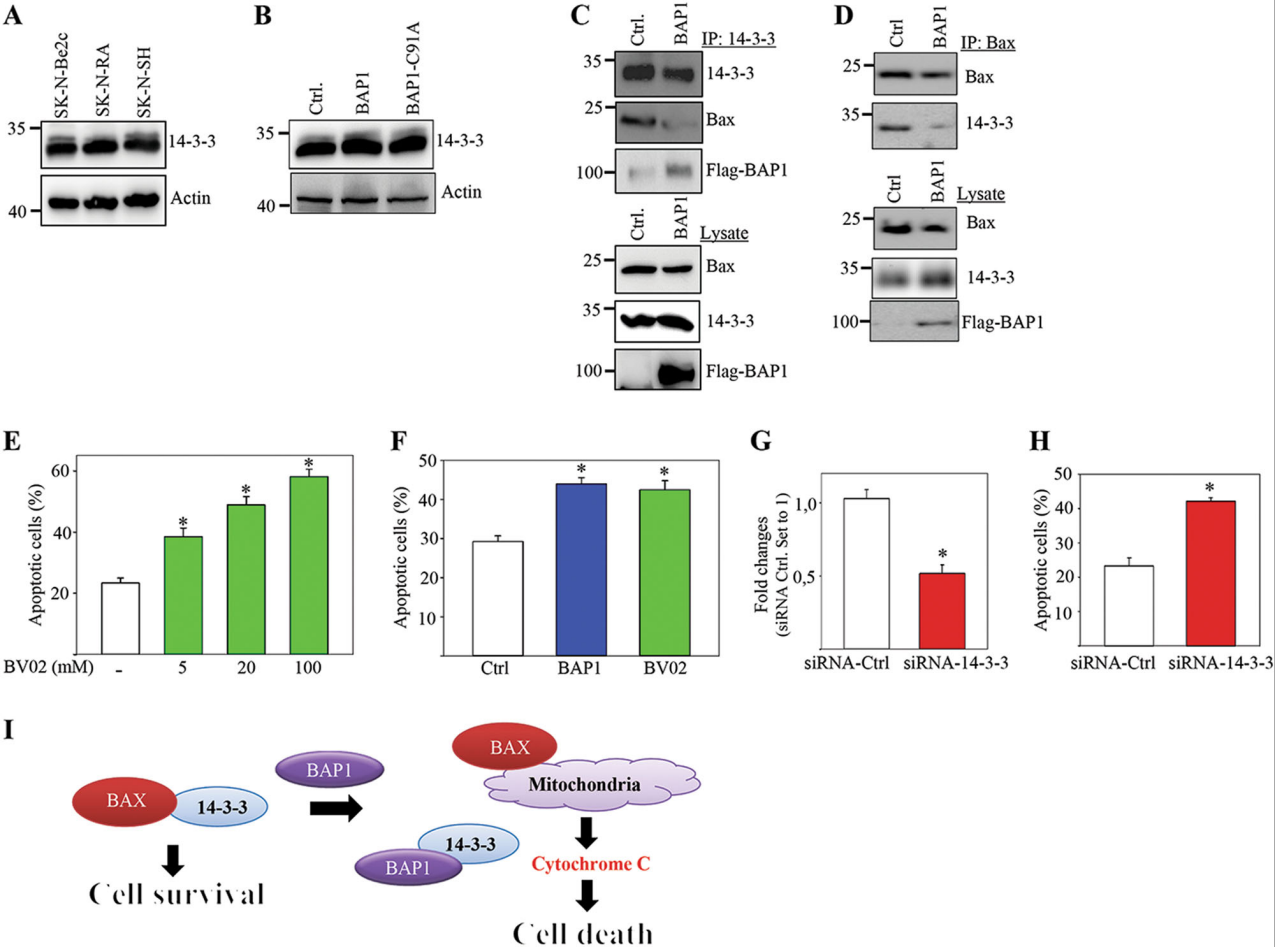
BAP1 binding to 14-3-3 releases Bax for inducing apoptosis. **a** Western blot analysis of 14-3-3 and actin expression in SK-N-Be2c, SK-N-SH, and SK-N-RA cells (*n* = 5). **b** Western blot analysis of 14-3-3 and actin expression in SK-N-Be2c transiently transfected with empty vector, full-length BAP1, or catalytically inactive mutant of BAP1 (BAP1-C91A) (*n* = 4). **c**, **d** SK-N-RA cells were transfected with FLAG-tagged full-length BAP1 or FLAG-empty vector. The lysates were immunoprecipitated with antibodies against 14-3-3 (**c**) or Bax (**d**) and probed with antibodies against 14-3-3 or Bax as indicated in the figure (*n* = 3). **e** Titration of BV02 in SK-N-RA cells for 24 h using three different concentrations (5, 20, and 100 µM). The percentage of apoptotic cells is presented as four independent experiments in triplicate (*p < 0.05). **f** SK-N-RA cells were transfected with control expression plasmid (white), transfected with full-length BAP1 expression plasmid (blue), or transfected with control expression plasmid and treated with 20 µM BV02 for 24 h (green). The percentage of apoptotic cells is presented as three independent experiments in triplicate (*p < 0.05). **g** Relative 14-3-3-ζ mRNA expression levels as fold changes measured using real-time reverse transcription PCR (qRT-PCR) of cDNA from transfected SK-N-RA cells with siRNA against 14-3-3-ζ or siRNA-GFP-Ctrl. The data are represented as three independent experiments (*p < 0.05). **h** The percentage of apoptotic cells in SK-N-RA cells transfected with siRNA against 14-3-3-ζ or siRNA-GFP-Ctrl in four independent experiments (*p < 0.05). **i** Model of BAP1-induced regulation of cell survival and cell death in neuroblastoma cells. BAP1 induces cell death via an interaction with 14-3-3 protein. The association between BAP1 and 14-3-3 releases the apoptotic inducer protein Bax from 14-3-3 and promotes cell death through the intrinsic (mitochondria) apoptosis pathway

Seeking an explanation for BAP-mediated apoptosis and the reduced expression of cell survival factors in neuroblastoma cells, we performed a LC–MS/MS analysis. Immunoprecipitation (IP) of FLAG-tagged BAP1 in SK-N-SH followed by LC–MS/MS analysis identified different isoforms of 14-3-3 proteins including 14-3-3-σ, -ζ, -ε, and -β (Fig. 3a, Supplementary Fig. 11, and Supplementary Table 1). Figure 3a shows the LC–MS/MS analysis and one example of a tandem mass spectrum that unambiguously identifies the sequence 191–224 of 14-3-3-σ. Further, for the protein 14-3-3-σ, 23 peptides corresponding to 83% of sequence coverage were identified (Supplementary Fig. 11 and Supplementary Table 1). Among the other previously identified BAP1 interacting partners (https://string-db.org/), we could confirm binding to CBX3, IPO4, IPO5, and RBBP7 (Supplementary Fig. 12). The interaction between BAP1 and 14-3-3 protein was confirmed by performing the co-IP of FLAG-tagged BAP1 and pull-down of endogenous 14-3-3 in neuroblastoma cells (Fig. 3b). The reciprocal IP using 14-3-3 antibodies also indicated the formation of the complex between BAP1 and 14-3-3 (Fig. 3c). In addition, fluorescence resonance energy transfer (FRET) assay demonstrated a direct interaction between BAP1 and 14-3-3 (Fig. 3d). GST pull-down experiments demonstrated that BAP1 could associate with all of the 14-3-3 isoforms in the cell (Fig. 3e). To define which domain(s) in BAP1 is required for interaction with 14-3-3-σ, a series of FLAG-tagged BAP1 deletion mutants (Fig. 3f) were transiently transfected together with full-length GST-tagged 14-3-3-σ into HEK293T cells. GST pull-down assays demonstrated that the deletion mutants containing the BAP1 domains NORS (non-regular secondary structure), and ULD (ubiquitin-like domain) interact with 14-3-3-σ (Fig. 3g and Supplementary Fig. 13A). Furthermore, GST pull-down assays using 14-3-3-σ deletion mutants (Fig. 3g), showed an interaction between truncation 163-248 and full-length BAP1 (Fig. 3h and Supplementary Fig. 13B). We could also confirm that the short fragment of BAP1-ULD domain and 14-3-3-alpha helices 7-9 regions facilitate interaction between these two proteins (Fig. 3i), while the interaction between BAP1 and 14-3-3-σ inactive mutants were reduced compared to the full-length 14-3-3-σ (Fig. 3j). We could not observe any differences in the total levels of 14-3-3 protein expression in the cells expressing low levels of BAP1 (Fig. 4a) or cells that have been transfected with full-length BAP1 or BAP1-C91A (Fig. 4b). To analyze, whether 14-3-3 can undergo ubiquitination, the overall levels of ubiquitin-associated 14-3-3 in BAP1- or empty plasmid-transfected neuroblastoma was investigated. We could not observe any differences in the levels of 14-3-3 ubiquitination in the absence or presence of BAP1 expression (Supplementary Fig. 14). The overexpression of BAP1-C91A did not show any differences in the 14-3-3 ubiquitination compared to the BAP1 expressing cells (Fig. 4b and Supplementary Fig. 14). Next, we tested the hypothesis that the BAP1 interaction with 14-3-3 releases the binding between 14-3-3 and Bax, which further facilitates cell death. Indeed, an IP experiment, showed that the interaction between 14-3-3 and Bax is abrogated in the presence but not in the absence of BAP1 (Fig. 4c). The reciprocal IP using Bax antibodies confirmed the release of the interaction between Bax and 14-3-3 in the presence of BAP1 (Fig. 4d). The treatment of neuroblastoma cells with BV02, which is an inhibitor of 14-3-3 and prevents the scaffolding function of 14-3-3 promoted cell death in a concentration-dependent manner (Fig. 4e). The percentage of apoptotic cells treated with 20 µM BV02 was similar to the cells transfected with BAP1 (Fig. 4f). In addition, siRNA oligos protected neuroblastoma cells against 14-3-3-ζ facilitated cell death (Fig. 4g, h). These results suggest that BAP1 interaction with 14-3-3 prevents cell survival signaling, which is essential for growth of the neuroblastoma cells (Fig. 4i).

**Fig. 5:**
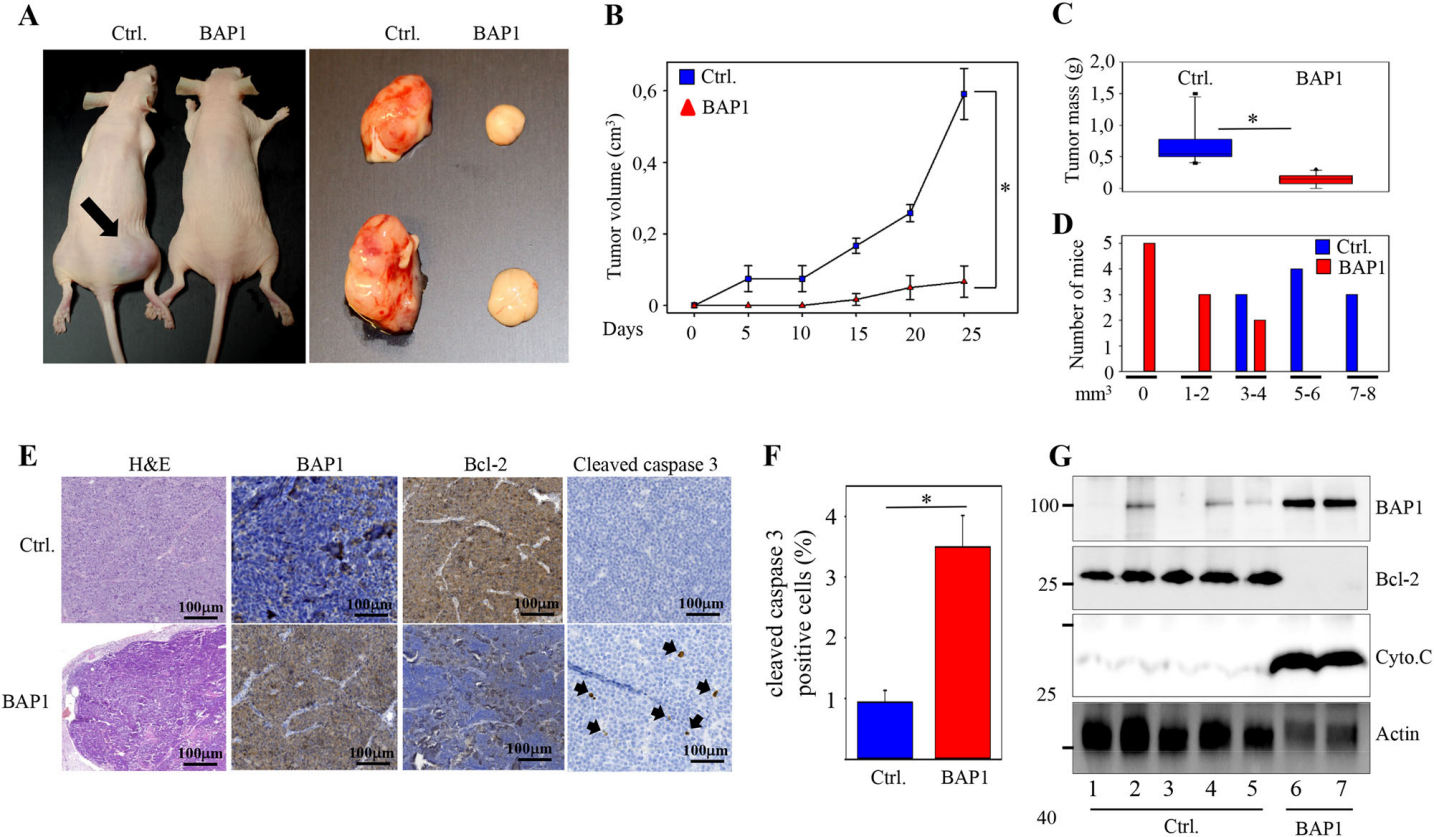
BAP1 reduces tumor growth in vivo. **a**–**d** Nude mice were randomly divided into 2 groups of 10 mice after each being injected with 5 × 10^6^ SK-N-BE2c cells stably expressing empty expression vector (Ctrl) or FLAG-tagged full-length BAP1. Tumor sizes were measured at the indicated time points. Tumor volumes were estimated based on the length and width of the tumors: [mm^3^] = (length [mm]) × (width [mm])^2^ × 0.5. (V = L × W2 × 0.5). The number of animals with certain tumor volume is presented in **d**. The mean tumor volume for each treatment group was graphed plus/minus standard errors (*p < 0.05). **e** Immunohistochemistry analysis of tumor biopsies isolated from control (Ctrl) or BAP1 expressing neuroblastoma cells using H&E or antibodies against BAP1 (1:500), Bcl-2 (1:1000), and cleaved caspase-3 (Asp175, 1:500). Arrowheads indicate cleaved caspase-3 positive cells. **f** Quantification of the number of cleaved caspase-3 (Asp175) positive cells in percentage visualized in the sections isolated from control or BAP1 expressing neuroblastoma cells (*p* < 0.05*). **g** Relative BAP1 expression and its effect on the expression of Bcl-2 and cytochrome C was determined by western blot from tumor tissue. Actin was used as the internal controls for western blotting

Next, we subcutaneously implanted 5.0 × 10^6^ of control and BAP1 expressing neuroblastoma cells in nude mice and followed the growth of the tumor for 25 days. It was evident that the weight and mass of BAP1 stably expressing tumors were markedly reduced compared to control mice (Fig. 5a–d), confirming the effects of BAP1 on neuroblastoma cell growth in vivo. As expected, BAP1 expression was elevated in tumor cells infected with BAP1 compared with control cells using IHC and western blotting (Fig. 5e, g). In addition, the Bcl-2 expression in BAP1 tumor cells isolated from mice were reduced compared to the control cells in line with the elevated number of cleaved caspase-3 cells observed in BAP1 expressing tumor cells (Fig. 5e–g). Investigating the levels of cytochrome C in the tumor tissues isolated from animals revealed the downregulation of this protein in the control cells compared with BAP1 expressing tumors (Fig. 5g).

### BAP1 expression and copy number alterations in neuroblastoma patients and cell lines

**Fig. 6:**
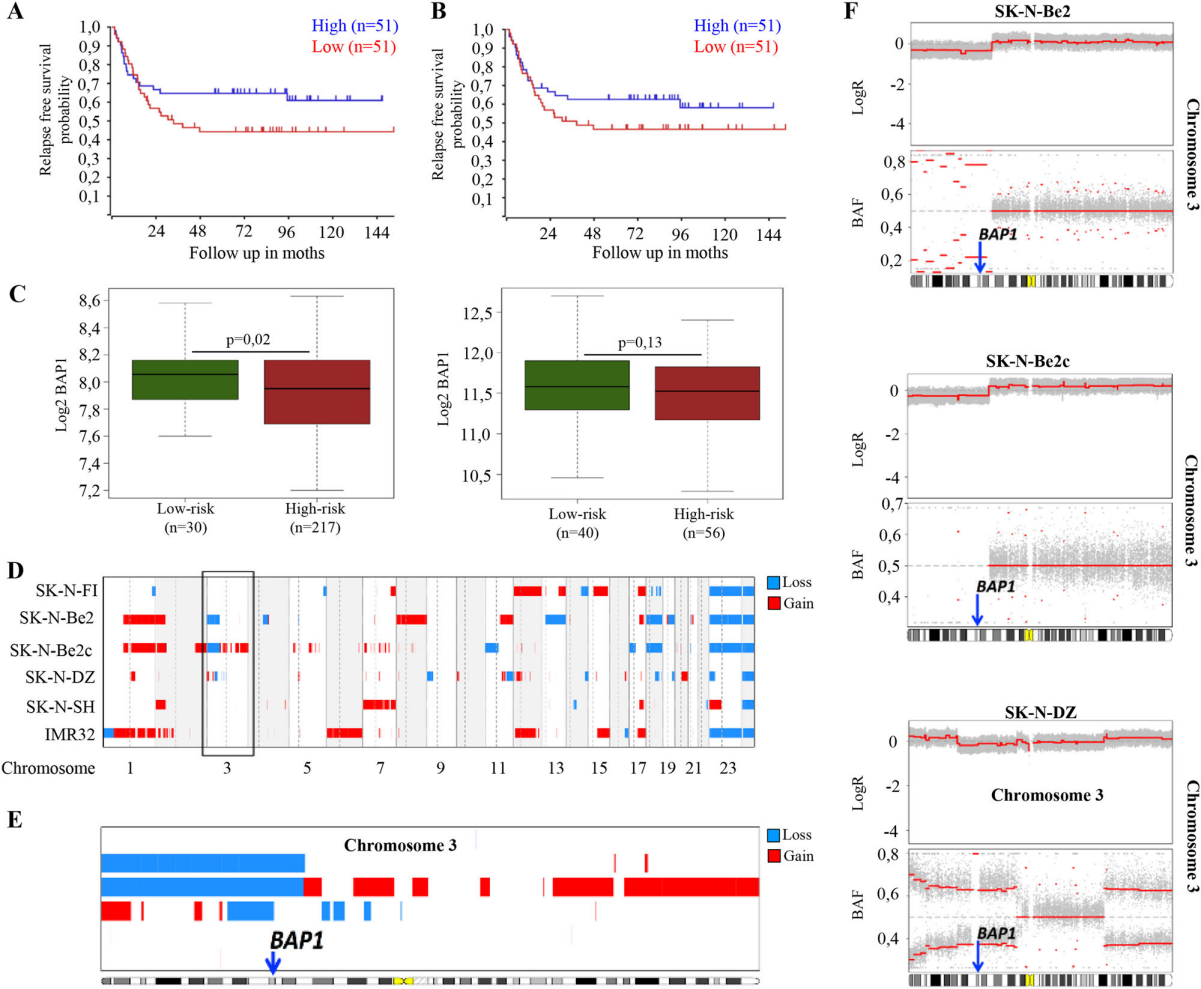
BAP1 expression and copy number alterations in neuroblastoma patients and cell lines. **a**, **b** BAP1 (*P* = 0.14) and Bax (*P* = 0.229) expression is associated with good outcome. Kaplan–Meier analysis using published array data from 102 patients with stage 4, non-MYCN amplified neuroblastoma. **c** Box plots showing the Log2 transformed expression profiles of BAP1 NB cohorts divided by COG Risk category (Intermediate risk patients were excluded). Left panel: BAP1 expression in the TARGET-NBL data set. Right panel: BAP1 expression in GSE73517 data. Gene expression data were downloaded from R2: Genomics Analysis and Visualization Platform (http://r2.amc.nl) and plotted using the R environment. Statistical significance was assessed by ANOVA test. **d** Whole-genome aberration plot of the six cell lines from the GEO series GSE89968 (in blue) are reported chromosomal losses and (in red) chromosomal gains, as well as the diploid regions in white. The black box highlights chromosome 3. **e** Zoomed-in view of chromosome 3 indicating altered regions. **f** Copy number alteration profile for chromosome 3 of SK-N-Be2c, SK-N-Be2c, and SK-N-DZ. LogR: log ratio values, BAF: B allele frequencies. The plots in **d**, **e** show deletion of BAP1 locus

The analysis of gene expression array data of 102 neuroblastomas (Stage 4 and non-MYCN amplified) showed that there is a trend toward the association between high BAP1 and Bax expression and better relapse-free survival of the patients (Fig. 6a, b). Furthermore, we evaluated BAP1 gene expression levels in two data sets of NB patients provided with COG risk category annotation. It was found lower levels of BAP1 expression in high-risk individuals of both data sets compared to low-risk patients (Intermediate risk patients were excluded, Fig. 6c). Copy number alterations profiles for SK-N-SH, SK-N-F1, IMR32, SK-N-DZ, SK-N-Be2, and SK-N-Be2c cell lines were obtained from recently published genotyping profiles^33,34^ by using Illumina HumanOmniExpress-24 v1.0 BeadChip (GPL21168).deposited at GEO database with accession numbers GSM2394365, GSM2394387, GSM2394364, GSM2394375, GSM2394372, and GSM2394383, respectively (GEO series: GSE89968). We used available log ratios and B allele frequencies to call copy number alterations with the R-Bioconductor package “copy number”^35^. The program was used to detect and remove outliers, impute missing values and to perform allele specific segmentation of SNP-array data. Log ratio thresholds for calling segments as gains or losses were set at 0.2 and −0.16, respectively. Figure 6d shows the whole-genome copy number alteration profile for the aforementioned cell lines. This analysis allowed us to confirm the well-known genomic features of the reported cell lines, including the loss-of-chromosome 1p in IMR32 and the amplification of MYCN in SK-N-DZ and IMR32. Figure 6e depicts a zoom-in of the copy number alterations profile for chromosome 3. We found that the cell line SK-N-Be2 had a large deletion (17,639,767 base pairs [bp], represented by 3870 probes on the chip) at chromosome 3p including BAP1 (Fig. 6f, upper panel) and shared with its descendant cell line SK-N-Be2c shown in Fig. 6f (middle panel). Further, we detected a homozygous deletion (Fig. 6f, lower panel), including chromosome 3p21.1 of SK-N-DZ cell line as reported in Cancer Cell Line Encyclopedia data^36^ available at cBioportal website (http://www.cbioportal.org). Here we used the Affymetrix Genome-Wide SNP 6.0 Array to estimate genomic copy number profiles and Affymetrix HG U133 Plus 2.0 platform to measure gene expression levels. Of the 1019 samples, we selected those for which mutation data, copy number profiles, and gene expression data were available (thus, restricting the analysis to 877 samples). Subjects that showed an alteration of BAP1 locus or its expression level numbered 148/877 or 17%. Copy number alteration profile for the SK-N-SH cell line treated with retinoic acid (SK-N-SH-RA) was available at the GEO repository (GSM999325, series: GSE40698) and at the UCSC Genome Browser as part of HAIB Genotype track set from ENCODE/HudsonAlpha (http://genome.ucsc.edu/cgi-bin/hgFileUi?db=hg19&g=wgEncodeHaibGenotype). No genomic alterations were found for the SK-N-SH cell line and for the derived SK-N-SH-RA cell line in chromosome 3p.

## Discussion

BAP1 is a tumor suppressor gene that functions as a deubiquitinating enzyme. The human BAP1 locus is located on chromosome 3p21.3. This region is commonly deleted or rearranged in many types of human cancer, such as lung, breast, melanoma, and renal cell carcinoma^13,37^. The role of BAP1 in neuroblastoma is unknown. In this study, we found that BAP1 was downregulated both at the RNA and protein level in a subset of neuroblastoma cell lines. Large scale hemizygous loss-of-chromosome 3p is a common event in neuroblastoma, and it is associated with tumors from older children^38,39^. Recurrent deletions have been found at locus 3p21-p22 in neuroblastoma and other common tumors, including breast and lung cancer, indicating that the same tumor suppressors are involved in tumorigenesis in these different tumors^40^. Deletion of chromosome 3p was also non-randomly associated with deletion of chromosome 11q in neuroblastoma^41^. Furthermore, loss-of-chromosomal material in chromosome arms 3p was discovered as a new high-risk subgroup of neuroblastoma stage 4 disease^38^. To investigate the role of BAP1, we overexpressed full-length BAP1 or BAP1-C91A in different neuroblastoma cell lines. The restoration of full-length BAP1 but not BAP1-C91A expression in these cells inhibited cell proliferation and promoted cell death suggesting that deubiquitin activity of BAP1 is necessary for the observed phenotype. More precisely, BAP1 increased the subG1 subpopulation of cells. Previous studies showed that BAP1 is known as a key regulator of cell cycle progression through G1/S transition^22,25^ and that BAP1 depletion in double-thymidine arrested HeLa cells leads to delayed entry into the S phase, potentially as a result of an inability of cells to respond to DNA damage during this phase^14^. Furthermore, in NCI-H226, which are BAP1-null cells, overexpression of wild-type BAP1 promotes G1 phase exit and subsequently speeds the transition to S phase^15^.

Two different strategies were applied to analyze the mechanism of BAP1 inducing cell death in neuroblastoma. First, we investigated the expression of apoptosis and survival target genes in cells expressing BAP1 compared to control cells. Second, we performed LC–MS/MS analysis to identify binding partners for BAP1 that could propagate cell death signaling. Gene expression profiling identified multiple genes in the cell survival pathway that were downregulated in BAP1 expressing cells. One of these target genes was the Bcl-2 protein. Independent of the neuroblastoma cell line tested, BAP1 expression promoted the downregulation of Bcl-2. The Bcl-2 family consists of anti-apoptotic, pro-apoptotic multi-domain, and pro-apoptotic BH3-only proteins. Bcl-2 and Bcl-XL are well known anti-apoptotic family members; whereas, Bax and Bak are among the pro-apoptotic multi-domain proteins^42^. In neuroblastoma, a large subset of patients harbor elevated levels of Bcl-2 compared to normal tissues^11^ and the silencing or inhibition of Bcl-2 in neuroblastoma cell lines results in apoptosis^11^. Further, siRNA targeting of Bcl-2 in neuroblastoma causes a high level of cell apoptosis and a significant suppression of tumor growth^43^. Previous studies also demonstrated that high-risk neuroblastoma cell lines derived from human tumors with the poorest prognosis are dependent on Bcl-2 for survival^10^. In addition, elevated Bcl-2 family protein expression was correlated to chemotherapy resistance^12^. In correlation with these previous findings, our data suggest that the expression of BAP1 leads to reduced expression of pro-survival factors such as Bcl-2, which in turn reduces the survival potential of these cells. Indeed, in vivo xenograft studies using BAP1 expressing cells showed reduced tumor growth compared to the control cells. Further, the tumors isolated from BAP1 expressing cells harbored an elevated number of cleaved caspase-3 and increased the expression of cytochrome C. In correlation to this result, the analyses of neuroblastoma patient tumor materials revealed that the high BAP1 and Bax mRNA expression shows a trend toward a better clinical outcome. Recent whole-genome sequencing studies of neuroblatoma did not find genomic aberrations of the BAP1 locus^44^. However, 3p deletion in neuroblastoma has been reported in previous studies^45^. Therefore, additional studies including treatment protocol data, protein expression, and larger sample sizes are necessary to investigate the potential role of the genomic aberrations of the BAP1 locus in driving the carcinogenesis of neuroblatoma.

LC–MS/MS analysis identified 14-3-3 proteins as novel binding partners for BAP1. The intracellular localization and scaffolding potential of 14-3-3 through direct interaction with their target protein is vital for regulating intracellular signaling pathways^46^. The 14-3-3 proteins can prevent cell apoptosis via direct interaction with Bcl-2 family members including Bad and Bax. In contrast, apoptotic stimulation, releases Bad or Bax from 14-3-3, and induces cytochrome C-mediated cell death^47^. The isoform specificity in our mass spectrometry analysis identified 14-3-3-σ, -ζ, -ε, and -β. Pull-down experiments and confocal microscopy demonstrated direct interaction and co-localization in the nucleus. Testing the total levels of 14-3-3 in the neuroblastoma cells, we found no changes in the cell lines harboring reduced levels of BAP1. In addition, we found no changes in the levels of 14-3-3 ubiquitination in the absence or presence of full-length BAP1 and BAP1-C91A. Instead, we found that BAP1 interaction with 14-3-3 releases the interaction between 14-3-3 and Bax, which in turn promotes cell death (Fig. 4i). The siRNA targeting of 14-3-3-ζ, or treating the cells with BV02, an inhibitor of 14-3-3 scaffolding protein docking sites, mimicked the effect of BAP1-mediated apoptosis. In general, the elevated levels of 14-3-3-ζ have been shown to be associated with different types of cancer, including lung, breast, prostate, myeloma, glioma, esophageal, head and neck, oral, pancreatic, ovarian, and skin. In most of these studies, the expression of 14-3-3-ζ was correlated with poor prognosis and chemoresistance^46,48^. In neuroblastoma BH3 peptides containing the domains of the pro-apoptotic BH3-only proteins Bid and Bad, -induced apoptosis and demonstrated antitumor efficacy in an in vivo model^49^.

In summary, our present findings suggest that the reduced expression of BAP1 triggers neuroblastoma cells to survive and grow faster compared to the BAP1 expressing cells. The restoration of BAP1 expression in neuroblastoma cells facilitated cell death mediated by direct interaction with 14-3-3 and releasing Bax from this complex. Our findings might have important implications for BAP1 to play a key role in the regulation of cell death in cancer cells, and the expression of BAP1 could have prognostic implications in predicting a good prognosis in cancer patients.

## Electronic supplementary material

Supplemental figures

Supplemental Table 1
