# Supplementary material for: BAP1 induces cell death via interaction with 14-3-3 in neuroblastoma
Source: Cell Death Dis. 2018 Apr 24;9(5):458. doi: 10.1038/s41419-018-0500-6 (PMC5913307; doi:10.1038/s41419-018-0500-6)
Supplement: Supplementary file 1 — Supplemental figures [file 41419_2018_500_MOESM1_ESM.pdf]

Suppl. Fig. 1

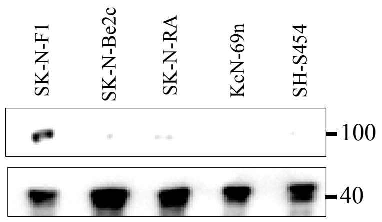

Supplementary figure 1. Western blot analysis of BAP1 and Actin expression in SK-N-F1, SK-N-Be2c, SK-N-RA, KcN-69n, SH-S454 (n=3).

Suppl. Fig. 2

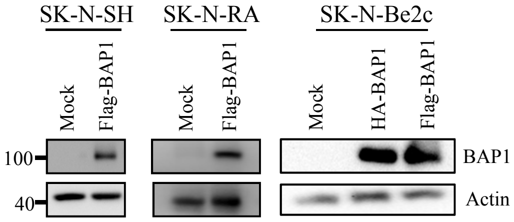

Supplementary figure 2. Western blot analysis of BAP1 and Actin expression in SK-N-SH, SK-N-RA, and SK-N-Be2c cells stably expressing Flag and HA-tagged full-length BAP1 (n=3).

Suppl. Fig. 3

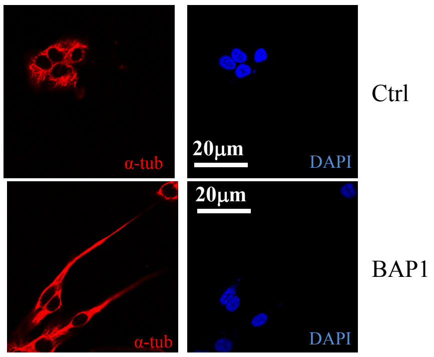

Supplementary figure 3. Cell morphology of neuroblstoma cells expressing BAP1 shows neurite outgrowth 96 hours post-transfection. The neurite outgrowth was analyzed by direct confocal microscopy using an anti-α-tubulin antibody (red, n=3).

Suppl. Fig. 4

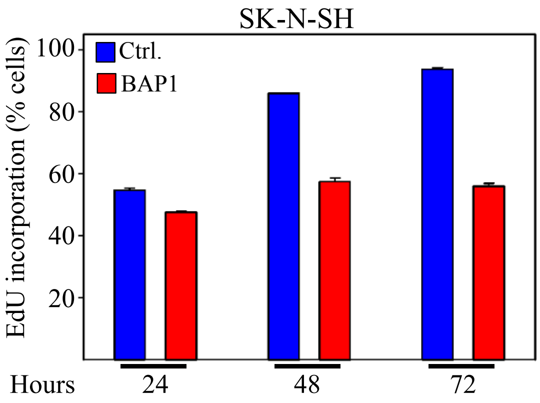

Supplementary figure 4. Bar graph shows EdU incorporation based proliferation analysis. SK-N-SH cells transfected with control or BAP1 expression plasmid were treated with 5μM EdU and after 24, 48 and 72 hours, the cells were collected and EdU incorporation was measured by FACS after Click-iT reaction was performed on fixed cells. EdU-unlabeled SK-N-SH cells were used to define the percentage of cell fractions with EdU incorporation (n=2).

Suppl. Fig. 5

	Ctrl.	BAP1	BAP1-C91A
SubG1	9,31± 2,3%	19,2± 5,5%	8,0± 3,4%
G1	62,6± 8,8%	34,7± 7,1%	59,6± 5,8%
S	13,7± 2,1%	22,1± 4,5%	11,7± 1,5%
G2/M	14,4± 3,7%	23,9± 5,5%	19,7± 6,1%

Supplementary figure 5. Changes in cell cycle distribution as a result of BAP1 or BAP1-mutant (BAP1-C91A) overexpression assessed using propidium iodide staining in unsynchronized SK-N-Be2c neuroblastoma cells (n=2).

Suppl. Fig. 6

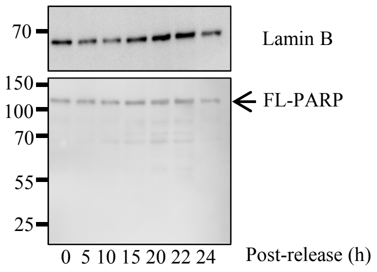

Supplementary figure 6. Extracts from synchronized and released SK-N-RA cells transfected with GFP control examined by western blotting in relation to post-release time points with an anti-PARP and anti-laminB. Arrowheads indicate FL-PARP: Full-length PARP (n=2).

Suppl. Fig. 7

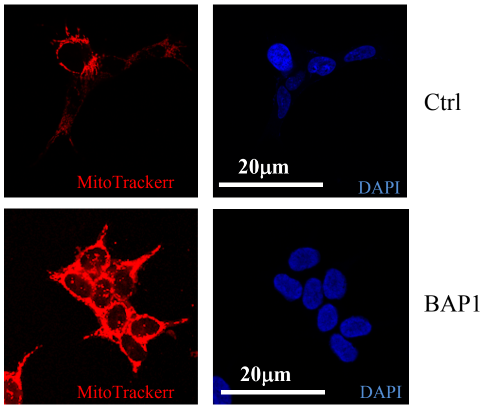

Supplementary figure 7. Release of Mito Tracker into the cytosol in BAP1 expressing cells compared to control SK-N-Be2c cells (Ctrl) following arsenic treatment (6 µM) examined by confocal microscopy (n=2).

Suppl. Fig. 8

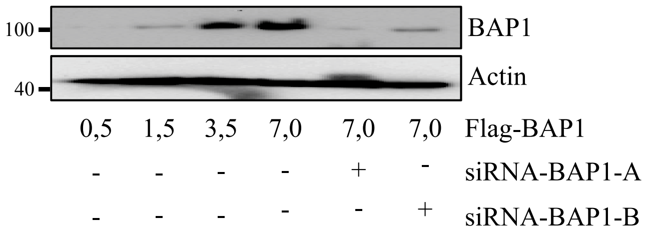

Supplementary figure 8. SK-N-BE2c cells were non-transfected (0 µg/µl) or transiently transfected with different concentration of full-length FLAG-tagged BAP1 expression plasmid (0.5 µg/µl, 1.5 µg/µl, 3.5 µg/µl, and 7.0 µg/µl) for 48 hours and transfected with siRNA oligos against BAP1 for another 48 hours as indicated in the figure.

Supplementary figure 5-8

Suppl. Fig. 9

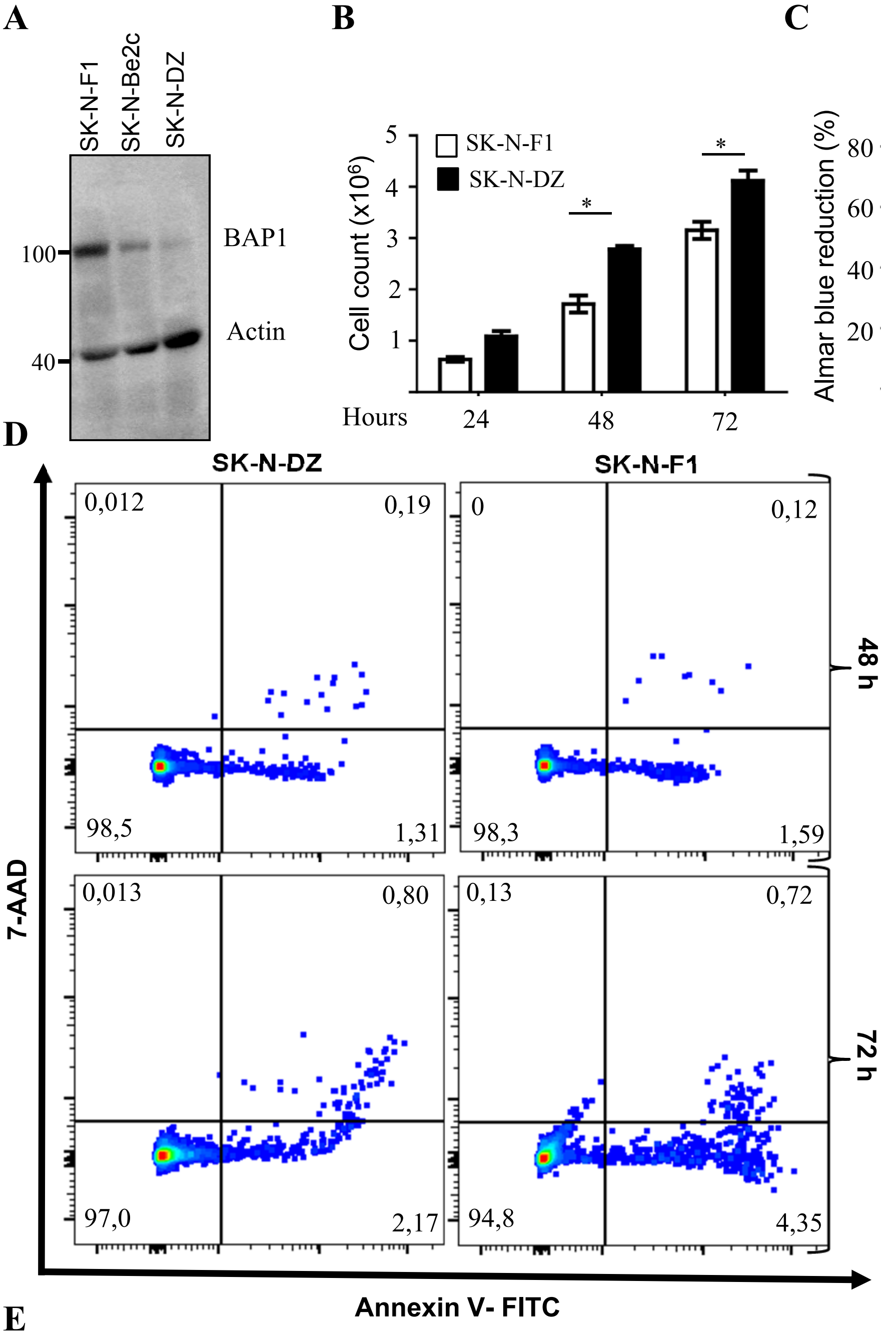

E

Cell line	Levels of BAP1	Number of mice	Number of mice with tumors
SK-N-Be2c	Low	16	15
SK-N-FI	High	10	1

Supplementary figure 9

(A) Western blot analysis of BAP1 and Actin expression from total cell lysates of SK-N-F1, SK-N-Be2c, and SK-N-DZ (n=2). **(B-C)** Direct total cell counts for SK-N-F1 and SK-N-DZ using the Countess™ automated cell counter and **(C)** cell proliferation was measured after 24, 48, and 72 hours using Alamar blue assay based on the quantitative metabolic conversion of blue, non-fluorescent resazurin to pink, fluorescent resorufin by living cells. The resazurin reduction in the cultures was determined after a 2-6 h incubation with Almar blue by measuring the absorbance at 530-nm and 590-nm wavelengths on Synergy-2 Multi-Mode Microplate Reader (Bio-tek Instruments). Data represent the average of at least two independent experiments performed in triplicates ± s.e.m. ($p < 0.05$*) **(D)** Representative experiment displaying the impact of endogenous BAP1 on apoptosis was examined in SK-N-DZ (BAP1-low) and SK-N-F1 (BAP1-high) cells by flow cytometry after 48 h and 72 h following PE and 7-AAD staining. Quadrants are as follows: lower left: viable cells; lower right: early apoptotic; upper right: late apoptotic/necrotic. Numbers in quadrants represent the percentage of the viable cells (lower left), early apoptotic (lower right), and late apoptotic/necrotic (upper right, n=2). **(E)** Nude mice were divided into 2 groups after each being injected with 5×10⁶ SK-N-Be2c or SK-N-FI cells. Table the number of animals with tumor after 25 days post xenograft transplantation.

Suppl. Fig. 10

A

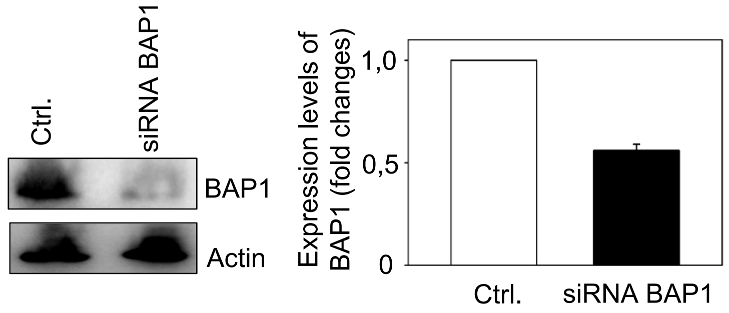

B

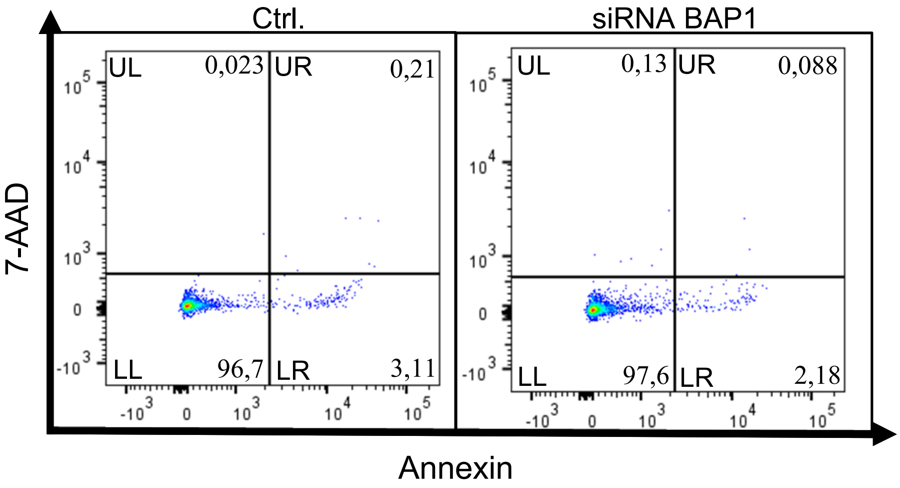

C

	Ctrl.	siRNA BAP1
Living cells (LL)	97,3 ± 0,3%	97,6 ± 0,05%
Early apoptotic cells (LR)	3,11 ± 0,34%	2,18 ± 0,02%
Late apoptotic cells (UR)	0,21 ± 0,005%	0,088 ± 0,01%

Supplementary figure 10. (A) Western blot analysis of BAP1 and Actin in BAP1-siRNA or control treated SK-N-F1 cells. (Left panel). Expression levels of BAP1 as fold changes in SK-N-FI cells transfected with control siRNA or siRNA against BAP1.The data are represented as two independent experiments. **(B)** SK-N-F1 cells were transiently transfected with siRNA oligos against BAP1 or with control siRNA and analyzed for apoptosis by flow cytometry following PE and 7-AAD staining. Quadrants are as follows: lower left: viable cells; lower right: early apoptotic; upper right: late apoptotic/necrotic. Numbers in quadrants represent the percentage of the viable cells (lower left), early apoptotic (lower right), and late apoptotic/necrotic (upper right). **(C)** The table shows the distribution of viable cells, early apoptotic and late apoptotic/necrotic from three independent experiment.

Suppl. Fig. 11

14-3-3-σ
score 297
83% sequence coverage
MERASLIQKA KLAEQAERYE DMAAFMKGAV EKGEELSCEE RNLLSVAYKN VVGGQRAAWR VLSSIEQKSN EEGSEEKGPE VREYREKVET ELQGVCDTVL GLLDSHLIKE AGDAESRVFY LKMKGDYYRY LAEVATGDDK KRIIDSARSA YQEAMDISKK EMPPTNPIRL GLALNFSVFH YEIANSPEEA ISLAKTTFDE AMADLHTLSE DSYKDSTLIM QLLRDNLTLW TADNAGEEGG EAPQEPQS

14-3-3-ζ
score 262
69% sequence coverage
MDKNELVQKA KLAEQAERYD DMAACMKSVT EQGAELSNEE RNLLSVAYKN VVGARRSSWR VVSSIEQKTE GAEKKQQMAR EYREKIETEL RDICNDVLSL LEKFLIPNAS QAESKVFYLK MKGDYYRYLA EVAAGDDKKG IVDQSQQAYQ EAFEISKKEM QPTHPIRLGL ALNFSVFYYE ILNSPEKACS LAKTAFDEAI AELDTLSEES YKDSTLIMQL LRDNLTLWTS DTQGDEAEAG EGGEN

14-3-3-ε
score 177
45% sequence coverage
MDDREDLVYQ AKLAEQAERY DEMVESMKKV AGMDVELTVE ERNLLSVAYK NVIGARRASW RIISSIEQKE ENKGGEDKLK MIREYRQMVE TELKLICCDI LDVLDKHLIP AANTGESKVF YYKMKGDYHR YLAEFATGND RKEAAENSLV AYKAASDIAM TELPPTHPIR LGLALNFSVF YYEILNSPDR ACRLAKAAFD DAIAELDTLS EESYKDSTLI MQLLRDNLTL WTSDMQGDGE EQNKEALQDV EDENQ

14-3-3-β

score 183
50% sequence coverage
MTMDKSELVQ KAKLAEQAER YDDMAAAMKA VTEQGHELSN EERNLLSVAY KNVVGARRSS WRVISSIEQK TERNEKKQQM GKEYREKIEA ELQDICNDVL ELLDKYLIPN ATQPESKVFY LKMKGDYFRY LSEVASGDNK QTTVSNSQQA YQEAFEISKK EMQPTHPIRL GLALNFSVFY YEILNSPEKA CSLAKTAFDE AIAELDTLNE ESYKDSTLIM QLLRDNLTLW TSENQGDEGD AGEGEN

Supplementary figure 11. Immunoprecipitation of Flag-tagged BAP1 in SK-N-SH and LC-MS/MS analysis identified different isoforms of 14-3-3 proteins including 14-3-3-σ, -ζ, -ε, and -β. The protein 14-3-3-σ identified peptides (in red) corresponding to 83%, 14-3-3- ζ corresponding to 69% , 14-3-3- ε corresponding to 45% , and 14-3-3- β corresponding to 50% of sequence coverage.

Suppl. Fig. 12

CBX3
score 53
15% sequence coverage
MASNKTTLQK MGKKQNGKSK KVEEAEPEEF VVEKVLDRRV VNGKVEYFLK WKGFTDADNT WEPEENLDCP ELIEAFLNSQ KAGKEKDGTK RKSLSDSESD DSKSKKKRDA ADKPRGFARG LDPERIIGAT DSSGELMFLM KWKDSDEADL VLAKEANMKC PQIVIAFYEE RLTWHSCPED EAQ

IPO4
score 106
7% sequence coverage
MESAGLEQLL RELLLPDTER IRRATEQLQI VLRAPAALPA LCDLLASAAD PQIRQFAAVL TRRRLNTRWR RLAAEQRESL KSLILTALQR ETEHCVSLSL AQLSATIFRK EGLEAWPQLL QLLQHSTHSP HSPEREMGLL LLSVVVTSRP EAFQPHHREL LRLLNETLGE VGSPGLLFYS LRTLTTMAPY LSTEDVPLAR MLVPKLIMAM QTLIPIDEAK ACEALEALDE LLESEVPVIT PYLSEVLTFC LEVARNVALG NAIRIRILCC LTFLVKVKSK ALLKNRLLPP LLHTLFPIVA AEPPPGQLDP EDQDSEEEEL EIELMGETPK HFAVQVVDML ALHLPPEKLC PQLMPMLEEA LRSESPYQRK AGLLVLAVLS DGAGDHIRQR LLPPLLQIVC KGLEDPSQVV RNAALFALGQ FSENLQPHIS SYSREVMPLL LAYLKSVPLG HTHHLAKACY ALENFVENLG PKVQPYLPEL MECMLQLLRN PSSPRAKELA VSALGAIATA AQASLLPYFP AIMEHLREFL LTGREDLQPV QIQSLETLGV LARAVGEPMR PLAEECCQLG LGLCDQVDDP DLRRCTYSLF AALSGLMGEG LAPHLEQITT LMLLSLRSTE GIVPQYDGSS SFLLFDDESD GEEEEELMDE DVEEEDDSEI SGYSVENAFF DEKEDTCAAV GEISVNTSVA FLPYMESVFE EVFKLLECPH LNVRKAAHEA LGQFCCALHK ACQSCPSEPN TAALQAALAR VVPSYMQAVN RERERQVVMA VLEALTGVLR SCGTLTLKPP GRLAELCGVL KAVLQRKTAC QDTDEEEEEE DDDQAEYDAM LLEHAGEAIP ALAAAAGGDS FAPFFAGFLP LLVCKTKQGC TVAEKSFAVG TLAETIQGLG AASAQFVSRL LPVLLSTAQE ADPEVRSNAI FGMGVLAEHG GHPAQEHFPK LLGLLFPLLA RERHDRVRDN ICGALARLLM ASPTRKPEPQ VLAALLHALP LKEDLEEWVT IGRLFSFLYQ SSPDQVIDVA PELLRICSLI LADNKIPPDT KAALLLLLTF LAKQHTDSFQ AALGSLPVDK AQELQAVLGL S

IPO5
score 107
5% sequence coverage
MAAAAAEQQQ FYLLLGNLLS PDNVVRKQAE ETYENIPGQS KITFLLQAIR NTTAAEEARQ MAAVLLRRLL SSAFDEVYPA LPSDVQTAIK SELLMIIQME TQSSMRKKVC DIAAELARNL IDEDGNNQWP EGLKFLFDSV SSQNVGLREA ALHIFWNFPG IFGNQQQHYL DVIKRMLVQC MQDQEHPSIR TLSARATAAF ILANEHNVAL FKHFADLLPG FLQAVNDSCY QNDDSVLKSL VEIADTVPKY LRPHLEATLQ LSLKLCGDTS LNNMQRQLAL EVIVTLSETA AAMLRKHTNI VAQTIPQMLA MMVDLEEDED WANADELEDD DFDSNAVAGE SALDRMACGL GGKLVLPMIK EHIMQMLQNP DWKYRHAGLM ALSAIGEGCH QQMEGILNEI VNFVLLFLQD PHPRVRYAAC NAVGQMATDF APGFQKKFHE KVIAALLQTM EDQGNQRVQA HAAAALINFT EDCPKSLLIP LDNLVKHLH SIMVLKLQEL IQKGTKLVLE QVVTSIASVA DTAEEKFVPY YDLFMPSLKH IVENAVQKEL RLLRGKTIEC ISLIGLAVGK EKFMQDASDV MQLLLKTQTD FNDMEDDDPQ ISYMISAWAR MCKILGKEFQ QYLPVVMGPL MKTASIKPEV ALLDTQDMEN MSDDDGWEFV NLGDQQSFGI KTAGLEEKST ACQMLVCYAK ELKEGFVEYT EQVVKLMVPL LKFYFHDGVR VAAAESMPLL LECARVRGPE YLTQMWHFMC DALIKAIGTE PDSDVLSEIM HSFAKCIEVM GDGCLNNEHF EELGGILKAK LEEHFKNQEL RQVKRQDEDY DEQVEESLQD EDDNDVYILT KVSDILHSIF SSYKEKVLPW FEQLLPLIVN LICPHRPWPD RQWGLCIFDD VIEHCSPASF KYAEYFLRPM LQYVCDNSPE VRQAAAYGLG VMAQYGGDNY RPFCTEALPL LVRVIQSADS KTKENVNATE NCISAVGKIM KFKPDCVNVE EVLPHWLSWL PLHEDKEEAV QTFNYLCDLI ESNHPIVLGP NNTNLPKIFS IIAEGEMHEA IKHEDPCAKR LANVVRQVQT SGGLWTECIA QLSPEQQAAI QELLNSA

RBBP7
score 40
2% sequence coverage
MASKEMFEDT VEERVINEEY KIWKKNTPFL YDLVMTHALQ WPSLTVQWLP EVTKPEGKDY ALHWLVLGTH TSDEQNHLVV ARVHIPNDDA QFDASHCDSD KGEFGGFGSV TGKIECEIKI NHEGEVNRAR YMPQNPHIIA TKTPSSDVLV FDYTKHPAKP DPSGECNPDL RLRGHQKEGY GLSWNSNLSG HLLSASDDHT VCLWDINAGP KEGKIVDAKA IFTGHSAVVE DVAWHLLHES LFGSVADDQK LMIWDTRSNT TSKPSHLVDA HTAEVNCLSF NPYSEFILAT GSADKTVALW DLRNLKLKLH TFESHKDEIF QVHWSPHNET ILASSGTDRR LNVWDLSKIG EEQSAEDAED GPPELLFIHG GHTAKISDFS WNPNEPWVIC SVSEDNIMQI WQMAENIYND EESDVTTSEL EGQGS

Supplementary figure 12. Immunoprecipitation of Flag-tagged BAP1 in SK-N-SH and LC-MS/MS analysis identified different previously published BAP1 interacting partners including CBX3, IPO4, IPO5, and RBBP7. Identified peptides shows in red.

Supplementary figure 12

Suppl. Fig. 13

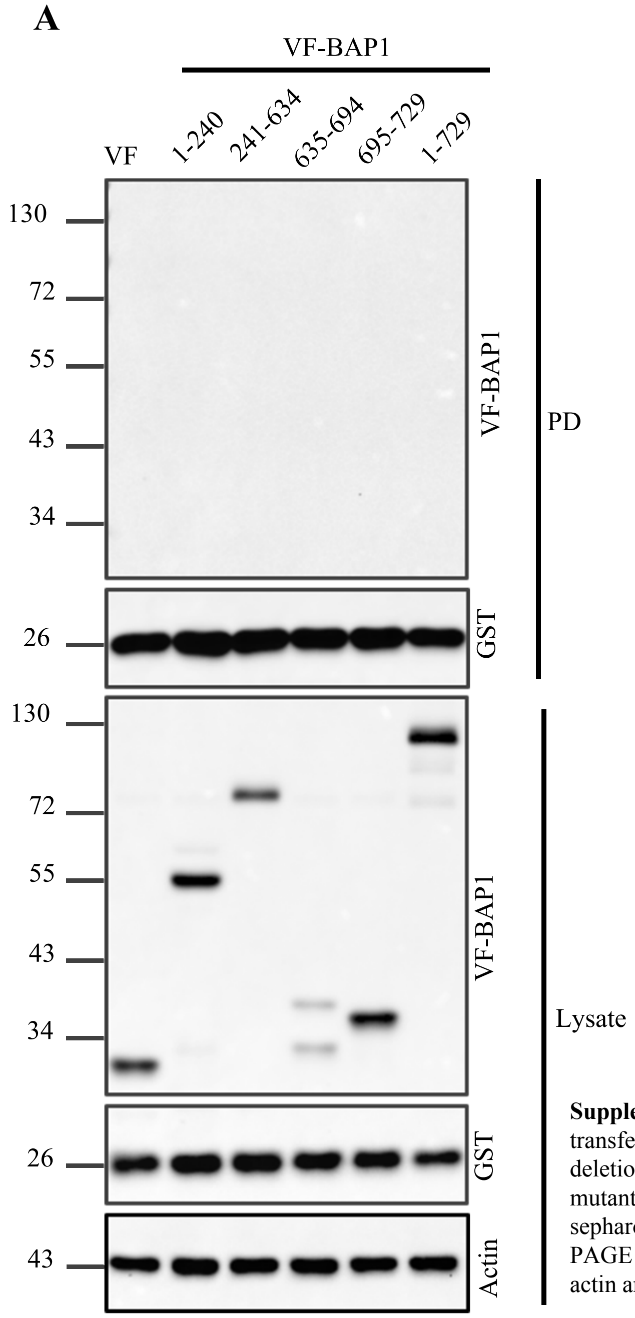

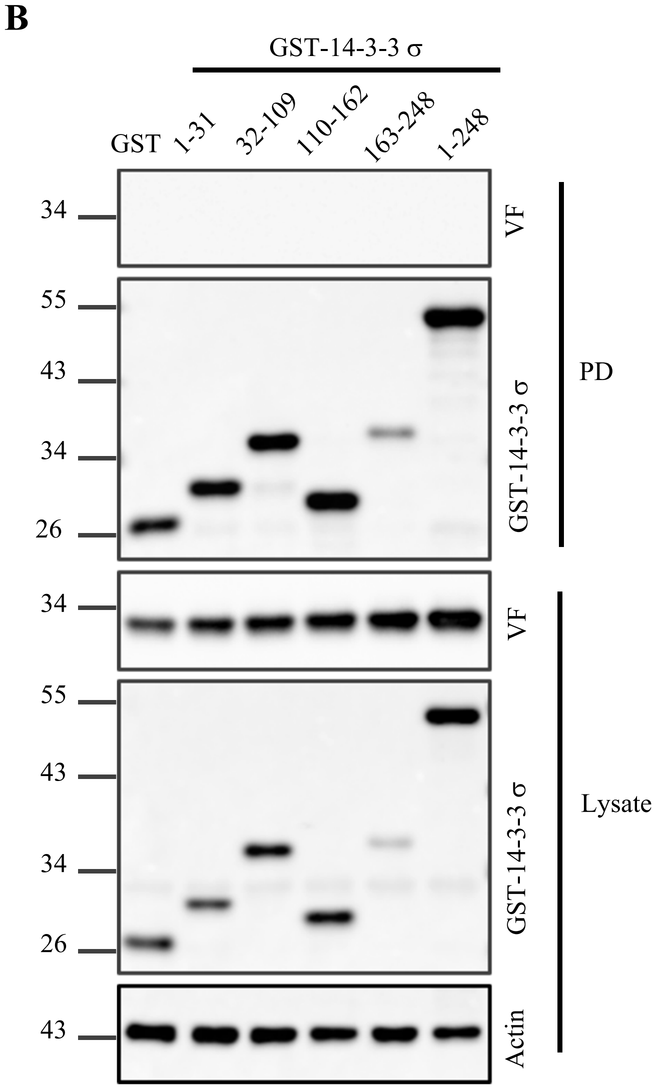

Supplementary figure 13. (A-B) GST pull-down experiment using co-transfection of cells with empty GST, VF-tagged full length and deletion mutants of BAP1 **(A)** or GST-tagged full length and deletion mutants of 14-3-3 σ **(B)** followed by addition of GST-conjugated sepharose beads. The eluted whole cell lysate was subjected to SDS-PAGE and analyzed by Western blot using anti-Flag, anti-GST, or anti-actin antibodies (n=3).

Suppl. Fig. 14

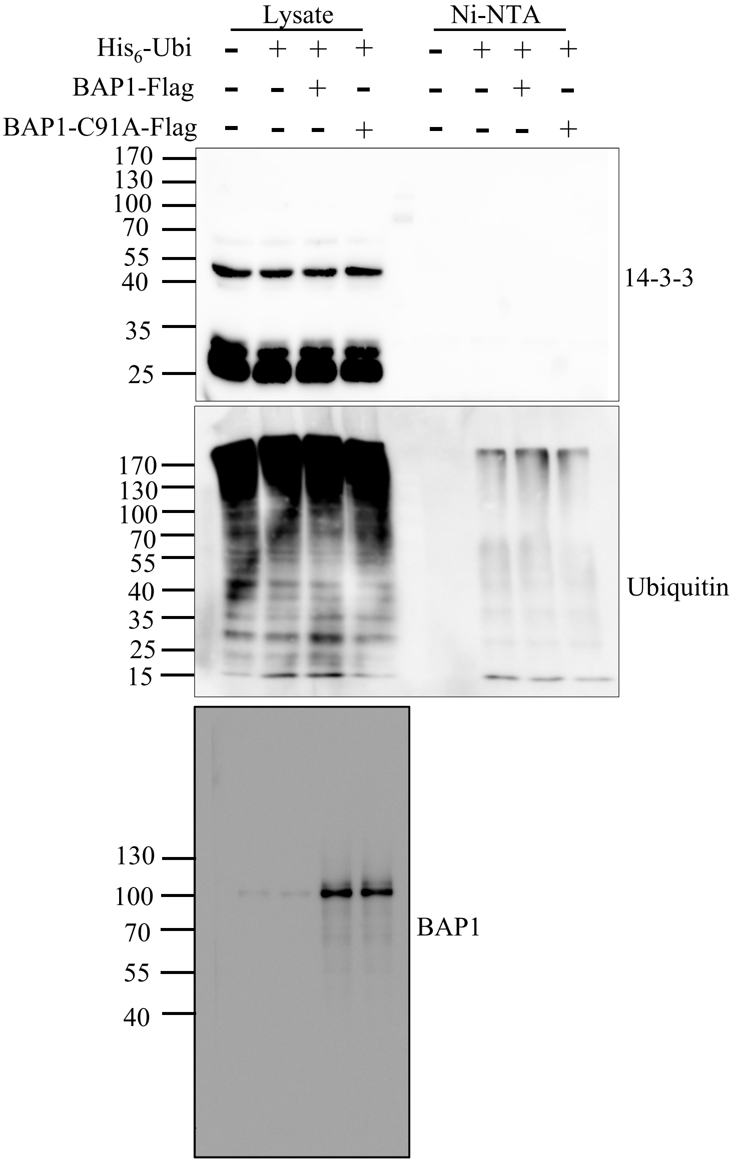

Supplementary figure 14. SK-N-RA cells were transfected with His6-Ubiquitin, Flag-tagged BAP1, BAP1-C91A or empty vector controls and lysates were subjected to His6/Ni-NTA chromatography. Isolated material was investigated with antibodies against 14-3-3 or ubiquitin. A tenth of total Ni-NTA purified material was blotted for ubiquitin (lower panel), while 1/2 of the total eluate was blotted for 14-3-3 (upper panel, n = 2).
