# Supplementary material for: BAP1 induces cell death via interaction with 14-3-3 in neuroblastoma
Source: Cell Death Dis. 2018 Apr 24;9(5):458. doi: 10.1038/s41419-018-0500-6 (PMC5913307; doi:10.1038/s41419-018-0500-6)
Supplement: Supplementary file 2 — Supplemental Table 1 [file 41419_2018_500_MOESM2_ESM.pdf]

```
Protein Accession,Peptide,Unique,-10lgP,Mass,Length,ppm,m/z,z,RT,Area,Fraction,Scan,#Spec,Start,End,PTM,AScore
P31947|1433S_HUMAN,K.TTFDEAMADLHTLSEDSYKDSTLIMQLLR.D,Y,92.93,3343.5952,29,-1.5,836.9048,4,70.8,4.61E+06,5,34167,2,196,224,,
P31947|1433S_HUMAN,R.EKVETELQGVC(+57.02)DTVLGLLDSHLIK.E,Y,76.95,2695.4102,24,-3.8,899.4739,3,64.15,2.26E+06,5,30631,2,86,109,Carbamidomethylation,C11:Carbamidomethylation:1000.00
P31947|1433S_HUMAN,K.TTFDEAM(+15.99)ADLHTLSEDSYKDSTLIMQLLR.D,Y,76.15,3359.5901,29,-2.6,840.9026,4,65.82,4.30E+06,5,31539,2,196,224,Oxidation (M),M7:Oxidation (M):125.43
P31947|1433S_HUMAN,R.LGLALNFSVFHYEIANSPEEAISLAK.T,Y,68.39,2832.4697,26,-3.1,945.1609,3,61.42,9.70E+06,5,29237,4,170,195,,
P31947|1433S_HUMAN,F.SVFHYEIANSPEEAISLAK.T,Y,66.62,2104.0476,19,0.1,702.3566,3,45.33,1.01E+06,5,20154,1,177,195,,
P31947|1433S_HUMAN,K.LAEQAERYEDMAAFMK.G,Y,66.06,1901.8651,16,-4.4,951.9357,2,41.63,,5,18195,2,12,27,,
P31947|1433S_HUMAN,K.TTFDEAMADLHTLSEDSYKDSTLIM(+15.99)QLLR.D,Y,65.33,3359.5901,29,-3.4,840.902,4,68.62,5.26E+06,5,33102,2,196,224,Oxidation (M),M25:Oxidation (M):74.24
P31947|1433S_HUMAN,R.SAYQEAMDISKK.E,Y,63.25,1369.6548,12,-2.1,457.5579,3,27.26,2.52E+07,5,10406,5,149,160,,
P31947|1433S_HUMAN,R.EKVETELQGVC(+57.02)DTVLGLLDSHLIKEAGDAESR.V,Y,61.96,3510.7512,32,-1.8,878.6935,4,61.36,4.86E+05,5,29108,1,86,117,Carbamidomethylation,C11:Carbamidomethylation:1000.00
P31947|1433S_HUMAN,K.SNEEGSEEKGPEVR.E,Y,61.43,1545.6907,14,-2.7,516.2361,3,19.19,6.30E+06,5,6115,2,69,82,,
P31947|1433S_HUMAN,K.DSTLIMQLLR.D,N,59.61,1188.6537,10,-1.3,595.3334,2,58.33,2.32E+06,5,27441,1,215,224,,
P31947|1433S_HUMAN,R.YEDMAAFMK.G,Y,59.57,1104.462,9,-1.1,553.2377,2,39.39,7.63E+06,5,16816,1,19,27,,
P31947|1433S_HUMAN,R.YLAEVATGDDK.K,Y,58.66,1180.5612,11,-1.6,591.2869,2,27.48,4.50E+06,5,10484,1,130,140,,
P31947|1433S_HUMAN,R.YLAEVATGDDKKR.I,Y,54.54,1464.7572,13,-3.8,489.2578,3,21.79,4.21E+06,5,7424,2,130,142,,
P31947|1433S_HUMAN,R.SAYQEAMDISK.K,Y,53.62,1241.5598,11,-3.7,621.7849,2,31.25,6.12E+06,5,12284,1,149,159,,
P31947|1433S_HUMAN,R.DNLTLWTADNAGEEGGEAPQEPQS,Y,51.96,2528.0938,24,-0.3,1265.0538,2,53.36,6.90E+06,5,24799,2,225,248,,
P31947|1433S_HUMAN,K.VETELQGVC(+57.02)DTVLGLLDSHLIK.E,Y,51.83,2438.2727,22,-4.2,813.7614,3,66.65,6.76E+06,5,32079,1,88,109,Carbamidomethylation,C9:Carbamidomethylation:1000.00
P31947|1433S_HUMAN,R.YLAEVATGDDKK.R,Y,51.64,1308.6561,12,-2.3,437.225,3,23.8,3.77E+05,5,8366,2,130,141,,
P31947|1433S_HUMAN,K.GAVEKGEELSC(+57.02)EER.N,Y,51.54,1591.7147,14,0.1,796.8647,2,23.07,1.72E+05,5,7991,1,28,41,Carbamidomethylation,C11:Carbamidomethylation:1000.00
P31947|1433S_HUMAN,K.LAEQAERYEDM(+15.99)AAFMK.G,Y,50.94,1917.86,16,-1.6,640.2929,3,34.85,6.07E+06,5,14251,2,12,27,Oxidation (M),M11:Oxidation (M):49.79
P31947|1433S_HUMAN,K.EMPPTNPIR.L,Y,50.64,1053.5277,9,-1.3,527.7704,2,27.21,4.35E+07,5,11542,2,161,169,,
P31947|14 M25:Oxidation (M):1000.00
P31947|1433S_HUMAN,K.GAVEKGEELSC(+57.02)EERNLLSVAYK.N,Y,45.57,2480.2217,22,-2,827.7462,3,41.59,,5,18040,1,28,49,Carbamidomethylation,C11:Carbamidomethylation:1000.00
P31947|1433S_HUMAN,K.GEELSC(+57.02)EERNLLSVAYK.N,Y,41.64,1995.9572,17,-3.5,666.324,3,42.76,1.93E+06,5,18708,1,33,49,Carbamidomethylation,C6:Carbamidomethylation:1000.00
P31947|1433S_HUMAN,K.GEELSC(+57.02)EER.N,Y,41.12,1107.4502,9,-0.9,554.7319,2,21.19,2.44E+05,5,7049,1,33,41,Carbamidomethylation,C6:Carbamidomethylation:1000.00
P31947|1433S_HUMAN,R.NLLSVAYK.N,N,40.89,906.5175,8,-1.6,454.2653,2,35.35,6.93E+07,5,14634,1,42,49,,
P31947|1433S_HUMAN,K.EM(+15.99)PPTNPIR.L,Y,39.17,1069.5226,9,-2.4,535.7673,2,23.75,4.55E+06,5,9608,1,161,169,Oxidation (M),M2:Oxidation (M):1000.00
P31947|1433S_HUMAN,R.YEDMAAFM(+15.99)K.G,Y,38.87,1120.4569,9,-0.9,561.2352,2,31,5.65E+05,5,12139,1,19,27,Oxidation (M),M8:Oxidation (M):28.74
P31947|1433S_HUMAN,K.LAEQAERYEDMAAFM(+15.99)K.G,Y,36.34,1917.86,16,-1.8,640.2928,3,36.27,3.45E+06,5,15050,1,12,27,Oxidation (M),M15:Oxidation (M):34.30
P31947|1433S_HUMAN,R.VLSSIEQK.S,N,35.09,902.5073,8,-2.2,452.2599,2,23.31,7.26E+06,5,8113,1,61,68,,
P31947|1433S_HUMAN,R.LGLALN(+.98)FSVFHYEIANSPEEAISLAK.T,Y,33.29,2833.4536,26,0.7,945.4924,3,65.46,,5,31504,1,170,195,Deamidation (NQ),N6:Deamidation (NQ):0.00
,,,,,,,,,,,,,,,,
Protein Accession,Peptide,Unique,-10lgP,Mass,Length,ppm,m/z,z,RT,Area,Fraction,Scan,#Spec,Start,End,PTM,AScore
P63104|1433Z_HUMAN,K.GIVDQSQQAYQEAFEISKK.E,Y,79.94,2168.075,19,-2.3,1085.0422,2,44.29,8.63E+05,5,19581,3,140,158,,
P63104|1433Z_HUMAN,K.SVTEQGAELSNEERNLLSVAYK.N,Y,77.33,2436.2131,22,-1.7,813.0769,3,46.55,1.47E+07,5,20851,1,28,49,,
P63104|1433Z_HUMAN,K.GIVDQSQQAYQEAFEISK.K,Y,73.3,2039.98,18,-0.9,1020.9964,2,48.76,3.74E+06,5,22090,2,140,157,,
P63104|1433Z_HUMAN,K.KGIVDQSQQAYQEAFEISK.K,Y,67.58,2168.075,19,1.8,723.7002,3,42.04,8.57E+05,5,18294,1,139,157,,
P63104|1433Z_HUMAN,K.SVTEQGAELSNEER.N,Y,64.88,1547.7063,14,-2.4,774.8586,2,25.62,3.62E+06,5,9397,1,28,41,,
P63104|1433Z_HUMAN,K.DSTLIMQLLR.D,N,59.61,1188.6537,10,-1.3,595.3334,2,58.33,2.32E+06,5,27441,1,213,222,,
P63104|1433Z_HUMAN,K.FLIPNASQAESK.V,Y,59.18,1303.6771,12,-1,652.8452,2,35.54,3.74E+07,5,14739,1,104,115,,
P63104|1433Z_HUMAN,R.DIC(+57.02)NDVLSLLEK.F,Y,56.61,1417.7123,12,-1.8,709.8621,2,63.12,6.49E+05,5,30067,1,92,103,Carbamidomethylation,C3:Carbamidomethylation:1000.00
P63104|1433Z_HUMAN,R.YLAEVAAGDDKK.G,Y,55.46,1278.6455,12,-1.8,427.2217,3,23.73,3.30E+06,5,8331,1,128,139,,
P63104|1433Z_HUMAN,K.LAEQAERYDDMAAC(+57.02)MK.S,Y,53.22,1900.8118,16,-1.1,634.6105,3,32.11,2.07E+06,5,12757,1,12,27,Carbamidomethylation,C14:Carbamidomethylation:1000.00
P63104|1433Z_HUMAN,K.TAFDEAIAELDTLSEESYK.D,Y,53.19,2130.9844,19,-4,711.3326,3,65.47,9.64E+05,5,31349,2,194,212,,
P63104|1433Z_HUMAN,R.YLAEVAAGDDKKGIVDQSQQAYQEAFEISKK.E,Y,50.27,3428.71,31,-0.4,686.749,5,43.19,1.07E+07,5,19058,1,128,158,,
P63104|1433Z_HUMAN,R.YLAEVAAGDDKKGIVDQSQ(+.98)QAYQEAFEISKK.E,Y,50.24,3429.6938,31,2.8,858.4331,4,43.07,,5,18882,1,128,158,Deamidation (NQ),Q19:Deamidation (NQ):0.00
P63104|1433Z_HUMAN,R.YLAEVAAGDDKKGIVDQSQQAYQEAFEISK.K,Y,46.35,3300.615,30,-3.9,826.1578,4,45.7,9.12E+05,5,20362,1,128,157,,
P63104|1433Z_HUMAN,K.IETELRDIC(+57.02)NDVLSLLEK.F,Y,41.5,2159.1143,18,-0.4,720.7117,3,64.24,2.51E+06,5,30682,1,86,103,Carbamidomethylation,C9:Carbamidomethylation:1000.00
P63104|1433Z_HUMAN,R.NLLSVAYK.N,N,40.89,906.5175,8,-1.6,454.2653,2,35.35,6.93E+07,5,14634,1,42,49,,
P63104|1433Z_HUMAN,R.EKIETELRDIC(+57.02)NDVLSLLEK.F,Y,37.99,2416.252,20,-0.5,605.0699,4,65.88,5.12E+05,5,31570,2,84,103,Carbamidomethylation,C11:Carbamidomethylation:1000.00
P63104|1433Z_HUMAN,K.TAFDEAIAELDTLSEESYKDST.L,Y,31.76,2434.0911,22,-0.7,812.3704,3,67.18,2.08E+05,5,32270,1,194,215,,
P63104|1433Z_HUMAN,R.DNLTLWTSDTQGDEAEAGEGGEN,Y,26.84,2407.9888,23,-1.2,1205.0002,2,53.22,9.23E+05,5,24718,1,223,245,,
P63104|1433Z_HUMAN,K.EMQPTHPIR.L,N,20.52,1107.5496,9,-2.3,554.7808,2,20.94,,5,6930,1,159,167,,
,,,,,,,,,,,,,,,,
Protein Accession,Peptide,Unique,-10lgP,Mass,Length,ppm,m/z,z,RT,Area,Fraction,Scan,#Spec,Start,End,PTM,AScore
P62258|1433E_HUMAN,K.AASDIAMTELPPTHPIR.L,Y,67.26,1818.9298,17,-0.9,607.3167,3,37.17,5.02E+06,5,15563,1,154,170,,
P62258|1433E_HUMAN,K.DSTLIMQLLR.D,N,59.61,1188.6537,10,-1.3,595.3334,2,58.33,2.32E+06,5,27441,1,216,225,,
P62258|1433E_HUMAN,R.YLAEFATGNDRK.E,Y,58.97,1383.6782,12,-2.2,462.2323,3,22.61,6.57E+06,5,8024,3,131,142,,
P62258|1433E_HUMAN,K.EAAENSLVAYK.A,Y,50.13,1193.5928,11,-2.3,597.8023,2,28.62,6.77E+06,5,10840,1,143,153,,
P62258|1433E_HUMAN,R.DNLTLWTSDMQGDGEEQNKEALQDVEDENQ,Y,46.28,3450.4641,30,-2.9,1151.1587,3,53.88,1.21E+06,5,24931,1,226,255,,
P62258|1433E_HUMAN,K.LAEQAERYDEMVESMKK.V,Y,42.84,2055.9604,17,-2.4,686.3258,3,34.19,,5,13881,1,13,29,,
P62258|1433E_HUMAN,R.NLLSVAYK.N,N,40.89,906.5175,8,-1.6,454.2653,2,35.35,6.93E+07,5,14634,1,43,50,,
P62258|1433E_HUMAN,R.QMVETELK.L,Y,32.92,976.4899,8,0.7,489.2526,2,26.3,2.69E+05,5,9659,1,87,94,,
P62258|1433E_HUMAN,K.VAGM(+15.99)DVELTVEER.N,Y,27.75,1462.6974,13,3.2,732.3583,2,34.04,1.04E+06,5,13793,1,30,42,Oxidation (M),M4:Oxidation (M):1000.00
P62258|1433E_HUMAN,R.YLAEFATGNDRKEAAENSLVAYK.A,Y,27.67,2559.2605,23,0.5,640.8227,4,36.63,,5,15253,1,131,153,,
P62258|1433E_HUMAN,K.AASDIAM(+15.99)TELPPTHPIR.L,Y,27.25,1834.9247,17,0.8,612.6494,3,33.11,,5,13308,1,154,170,Oxidation (M),M7:Oxidation (M):1000.00
,,,,,,,,,,,,,,,,
Protein Accession,Peptide,Unique,-10lgP,Mass,Length,ppm,m/z,z,RT,Area,Fraction,Scan,#Spec,Start,End,PTM,AScore
P31946|1433B_HUMAN,K.QTTVSNSQQAYQEAFEISKK.E,Y,64.64,2286.1128,20,-1.8,763.0435,3,34.91,2.92E+06,5,14286,1,141,160,,
P31946|1433B_HUMAN,K.DSTLIMQLLR.D,N,59.61,1188.6537,10,-1.3,595.3334,2,58.33,2.32E+06,5,27441,1,215,224,,
P31946|1433B_HUMAN,K.LAEQAERYDDMAAAMK.A,N,58.3,1811.8181,16,-1.7,604.9456,3,33.21,6.86E+06,5,13360,1,14,29,,
P31946|1433B_HUMAN,R.YLSEVASGDNK.Q,Y,55.05,1181.5564,11,-1.9,591.7844,2,24.59,1.60E+05,5,8776,1,130,140,,
P31946|1433B_HUMAN,K.TAFDEAIAELDTLNEESYK.D,Y,51.33,2157.9954,19,-0.6,720.3386,3,61.85,,5,29382,1,196,214,,
P31946|1433B_HUMAN,R.NLLSVAYK.N,N,40.89,906.5175,8,-1.6,454.2653,2,35.35,6.93E+07,5,14634,1,44,51,,
P31946|1433B_HUMAN,R.VISSIEQK.T,N,35.09,902.5073,8,-2.2,452.2599,2,23.31,7.26E+06,5,8113,1,63,70,,
P31946|1433B_HUMAN,R.YLSEVASGDNKQTTVSNSQQAYQEAFEISKK.E,Y,27.22,3449.6587,31,-0.4,863.4216,4,38.28,1.07E+07,5,16191,1,130,160,,
P31946|1433B_HUMAN,K.EMQPTHPIR.L,N,20.52,1107.5496,9,-2.3,554.7808,2,20.94,,5,6930,1,161,169,,
Q12907|LMAN2_HUMAN,K.DNFHGLAIFLDTYPNDETTER.V,Y,78.16,2467.1292,21,-1.7,823.3823,3,55.6,,5,25904,1,152,172,,
Q12907|LMAN2_HUMAN,R.LPTGYYFGASAGTGDLSDNHDIISMK.L,Y,69.03,2729.2642,26,0.1,910.7621,3,48.6,,5,21998,1,247,272,,
```
